# Supplementary figures and images for: Robust and consistent measures of pattern separation based on information theory and demonstrated in the dentate gyrus
Source: PLoS Comput Biol. 2024 Feb 20;20(2):e1010706. doi: 10.1371/journal.pcbi.1010706 (PMC10906873; doi:10.1371/journal.pcbi.1010706)

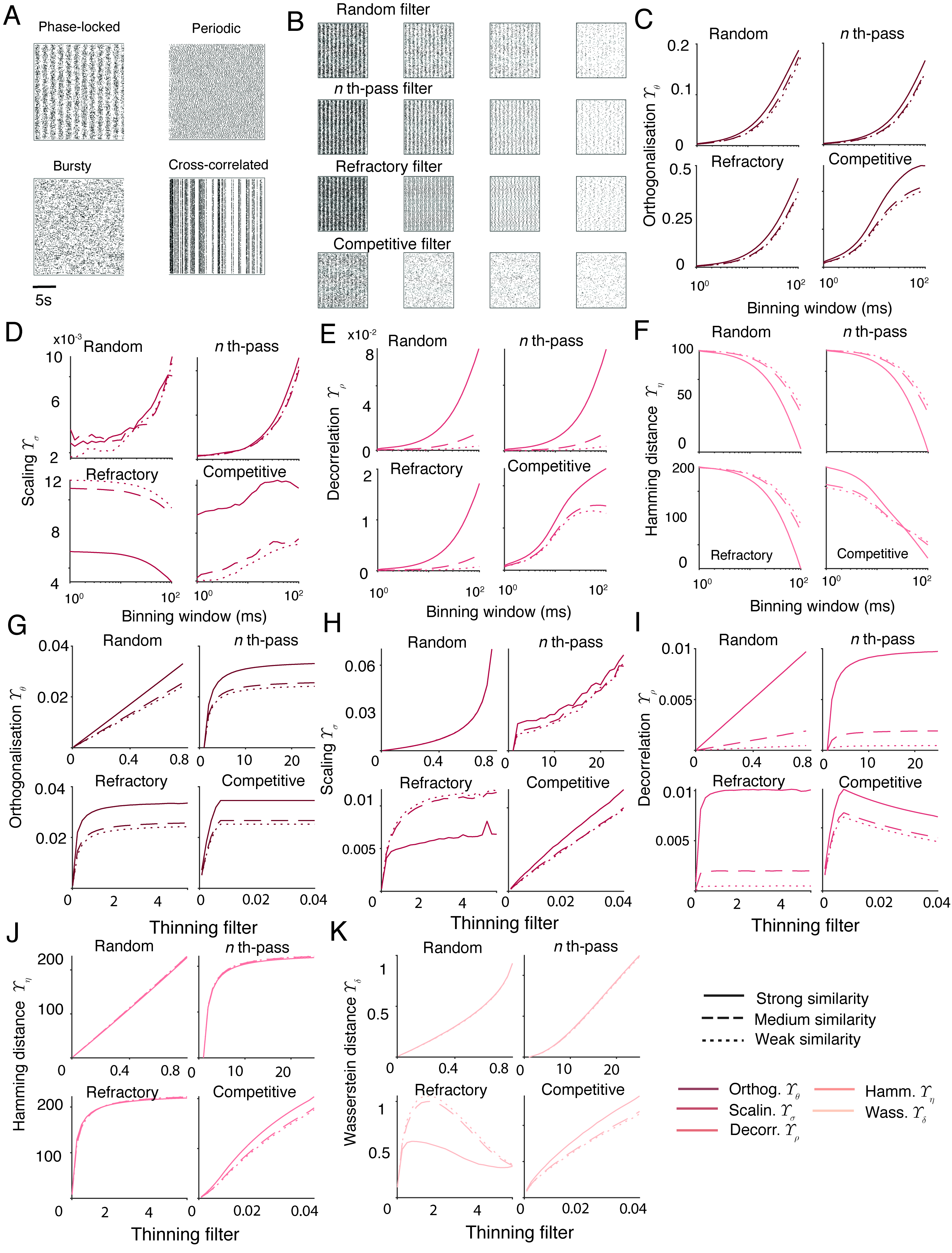

Supplement: S1 Fig — A Example rasters resulting from different methods of generating ensembles. All ensembles have a mean spike rate of 2.5Hz per neuron. Clockwise from top left: Phase-locked ensemble with a phase-locking strength of 0.9 and a phase rate of 0.6Hz, periodic ensemble with α = 10, cross-correlated ensemble with a similarity strength of 0.8, and bursty ensemble with α = 0.5. B Filtering of spike train ensembles. From top to bottom: random filtering with p = 0.5, 0.75, 0.85, 0.95, n-th pass filtering with n = 2, 4, 7, 20, refractory filtering with t = 0.18, 0.54, 1.01, 3.71 s, and competitive filtering with t = 3.4, 13.7, 17.9, 31.4 ms. Filtering parameters are chosen to give roughly equal numbers of spikes across different filters. C Unnormalised orthogonalisation Υθ values for comparison with Fig 1B. D Unnormalised scaling Υσ values for comparison with Fig 1B. E Unnormalised decorrelation Υρ values for comparison with Fig 1B. F Unnormalised Hamming distances Υη values for comparison with Fig 1B. G Unnormalised orthogonalisation Υθ values for comparison with Fig 1C. H Unnormalised scaling Υσ values for comparison with Fig 1C. I Unnormalised decorrelation Υρ values for comparison with Fig 1C. J Unnormalised Hamming distances Υη values for comparison with Fig 1C. K Unnormalised Wasserstein distances Υδ values for comparison with Fig 1C. (TIF) [file pcbi.1010706.s001.tif]

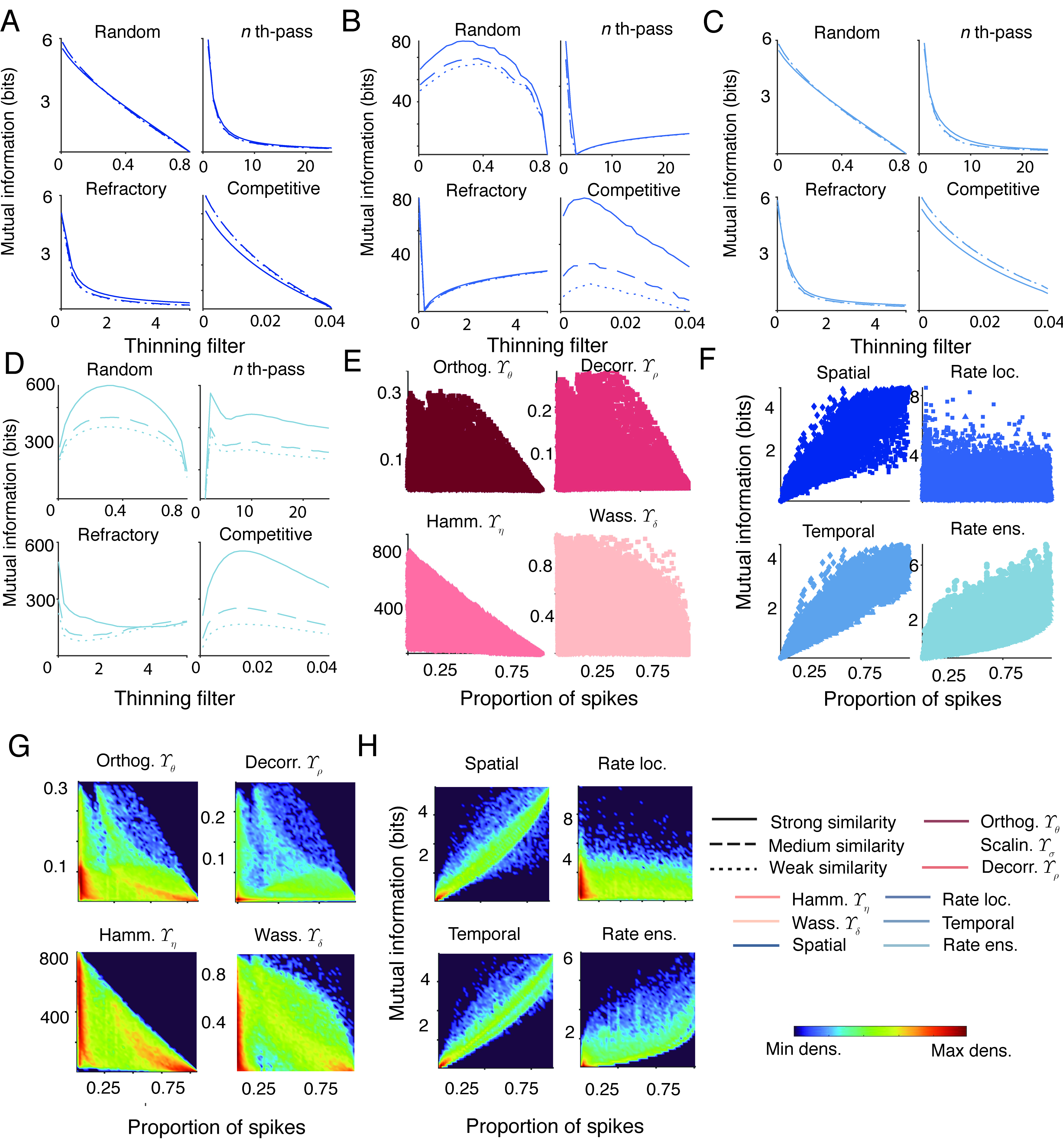

Supplement: S2 Fig — A Unnormalised mutual information values using the spatial code for comparison with Fig 1D. B Unnormalised mutual information values using the temporal code for comparison with Fig 1D. C Unnormalised mutual information values using the local rate code for comparison with Fig 1D. D Unnormalised mutual information values using the ensemble rate code for comparison with Fig 1D. Solid, dashed, and dotted lines in all panels refer respectively to strong, medium, and weak input similarities (see Methods). E Scatter plot of sparsity against different classical measures of pattern separation for comparison with Fig 1E. F Scatter plot of sparsity against mutual information computed using different codes for comparison with Fig 1E. Different markers correspond to different methods of ensemble generation: circles are for phase-locked ensembles, squares are for periodic ensembles, diamonds are for bursty ensembles, and triangles are for cross-correlated ensembles. G Density map corresponding to scatter points in E. H Density map corresponding to scatter points in F. Density values are logarithmic. There is a consistent negative relationship between the proportion of spikes that pass through the filter and pattern separation metrics. In the four cases, the linear correlations between the proportion of spikes passing through the system and the different measures are Υθ : −0.33, Υρ : −0.071, Υη : −0.68, and Υδ : −0.28. Conversely, the typical relationship between mutual information and the proportion of spikes is positive (Fig 1E), with linear correlations of 0.96 for the spatial code, 0.97 for the temporal code, 0.07 for the local rate code, and 0.73 for the ensemble rate code. (TIF) [file pcbi.1010706.s002.tif]

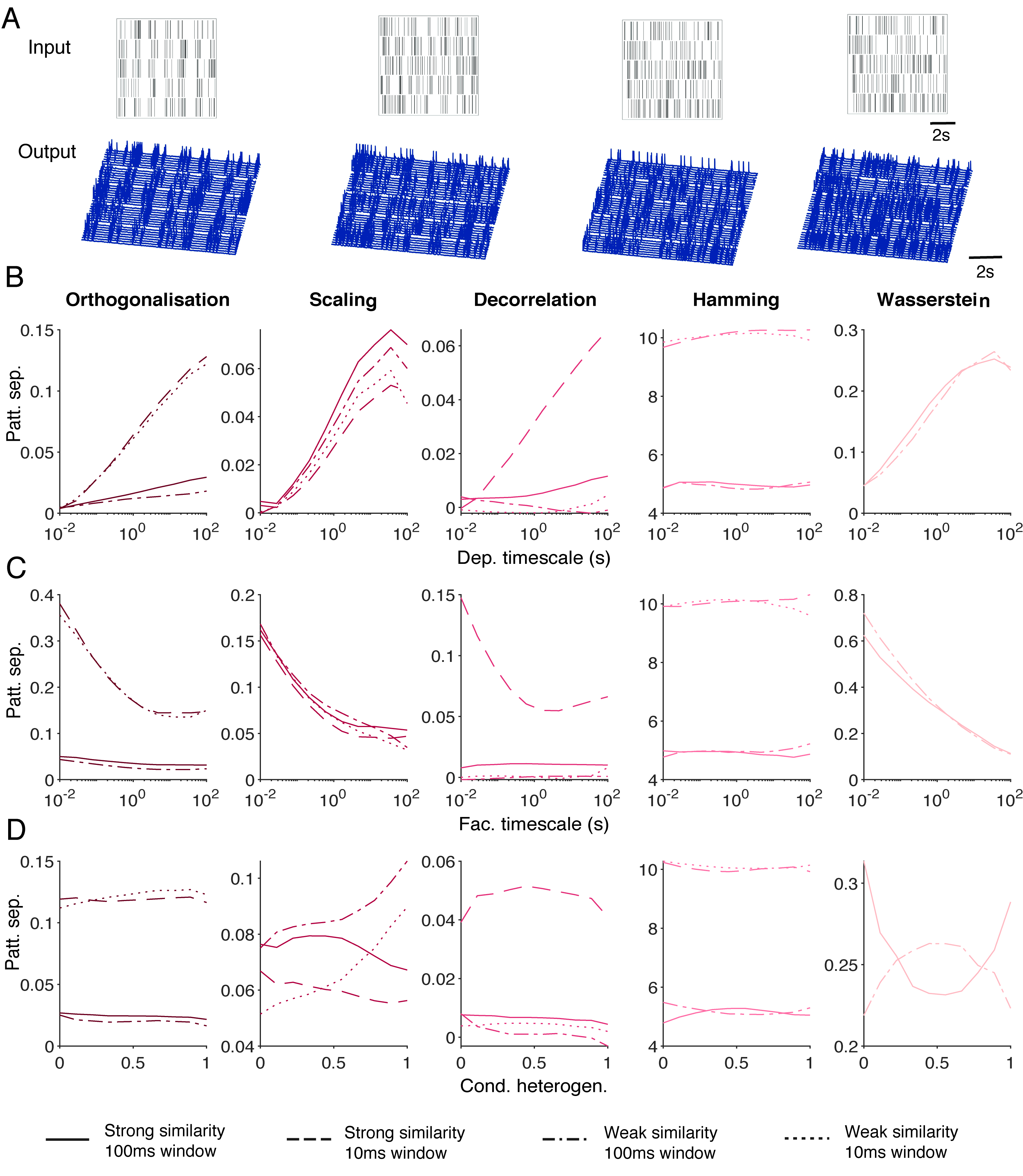

Supplement: S4 Fig — A Example input spiking rasters (top) and output voltage traces (bottom) for the single granule cell model. B Classical pattern separation measures applied to simulated voltage traces from a granule cell model with varied timescales of synaptic depression. From left to right: orthogonalisation Υθ, scaling Υσ, decorrelation Υρ, Hamming distance Υη, and Wasserstein distance Υδ. Solid lines correspond to a strong input similarity and a binning window (where applicable) of 100 ms, dashed lines correspond to a strong input similarity and a binning window of 10 ms, dash-dotted lines correspond to a weak input similarity and a binning window of 100 ms, and dashed lines correspond to a strong input similarity and a binning window of 10ms. C As above with varied timescales of synaptic facilitation. D As above with varied ion channel spatial heterogeneities. Input spike traces are two minutes long and consist of phase-locked inputs with a phase rate of 0.6Hz and a spiking rate of 5Hz. (TIF) [file pcbi.1010706.s004.tif]

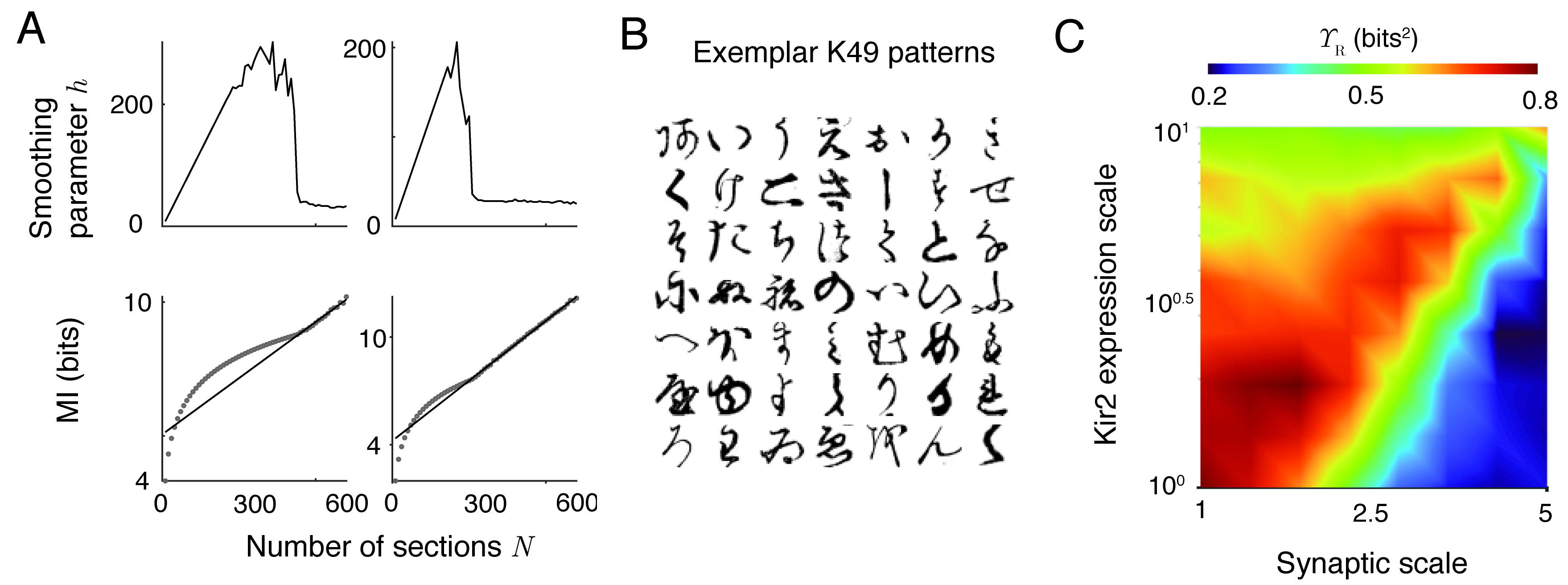

Supplement: S5 Fig — A Examples of extrapolating mutual information estimates. The left and right columns show two different ensembles. The top row shows the optimal smoothing parameter h (Eq 19) as a function of the number of sections the spike trains are divided into N. The bottom panels show the relationship between N and the mutual information estimate (grey circles), and the extrapolation back to infinitely long sections (black lines) from the region where h is significantly less than N and the relationship between N and the mutual information estimate is linear. B Exemplar patterns for each of the 49 classes in the Kuzushiji-49 dataset [101]. C Relative redundancy reduction ΥR for the mature granule cells as a function of scaling parameters for informative synapses and Kir2 channel expression. For comparison with Fig 5E. (TIF) [file pcbi.1010706.s005.tif]

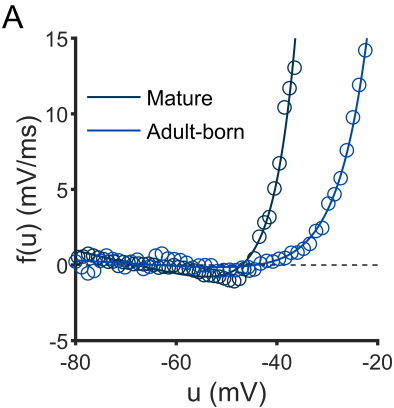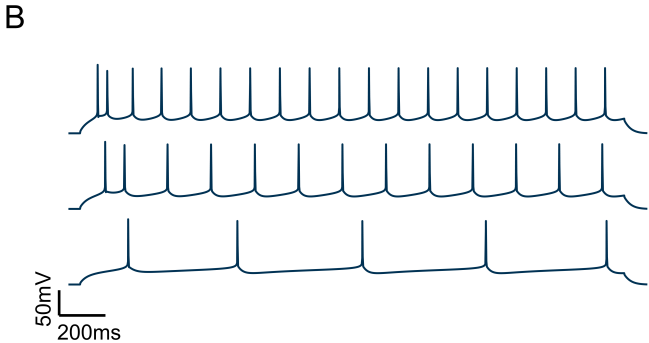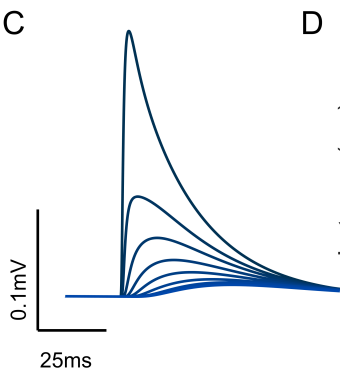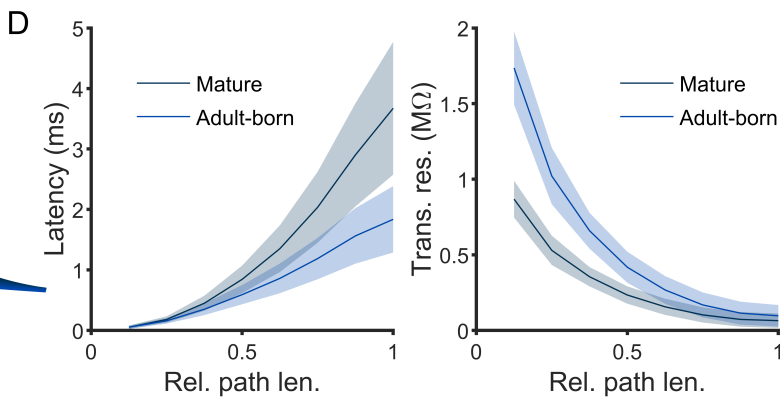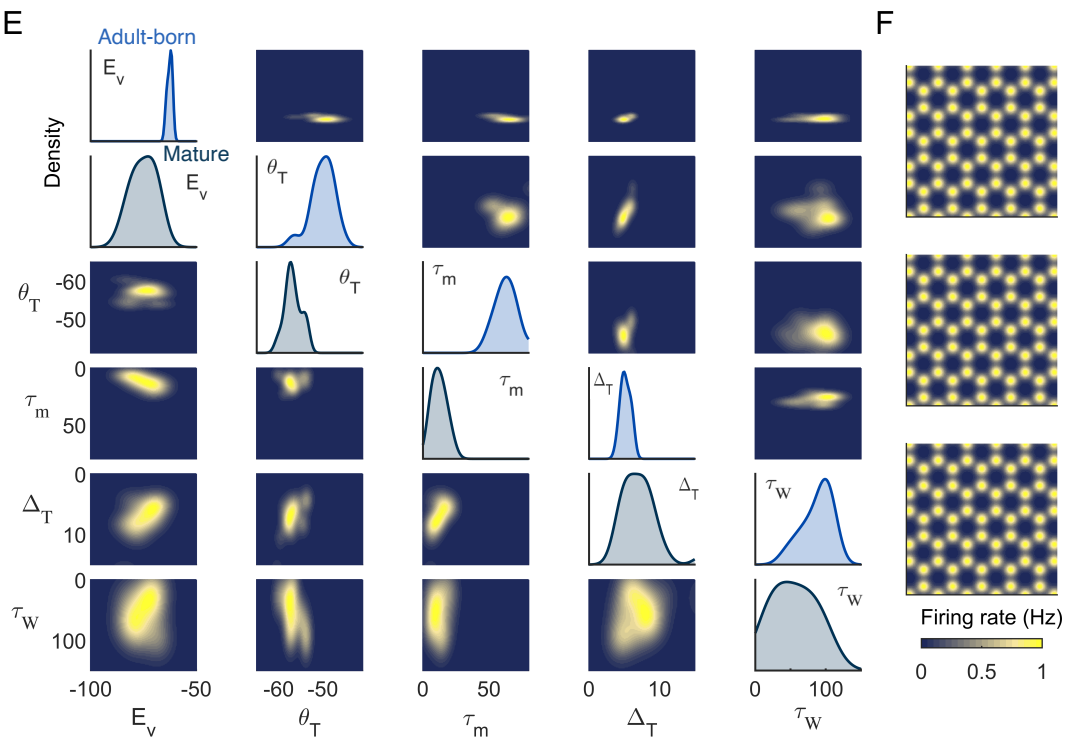

Supplement: S6 Fig — A Example dynamic IV curves for mature (dark blue) and adult-born (light blue) granule cells. Circles show the simulated results from full compartmental models and solid lines the best fit under Eq 24. B Example voltage traces for mature granule cells showing spike-frequency adaptation. C Example somatic voltages in a mature granule cell in response to synaptic inputs at increasing distances (lighter blue lines). D Latency of synaptic inputs at the soma (left) and transfer resistance to the soma (right) as a function of relative path length for mature (dark blue) and adult-born (light blue) granule cells. Shaded areas show one standard deviation around the mean. E Adaptive EIF parameter distributions for mature (lower left) and adult-born (upper right) granule cell compartmental models. The leading diagonal panels show single marginal distributions, and the off-diagonal panels show the pairwise marginals. F Examples of grid cell-like firing rates as a function of location (Eq 26). (PDF) [file pcbi.1010706.s006.pdf]
