# Supplementary material for: Robust and consistent measures of pattern separation based on information theory and demonstrated in the dentate gyrus
Source: PLoS Comput Biol. 2024 Feb 20;20(2):e1010706. doi: 10.1371/journal.pcbi.1010706 (PMC10906873; doi:10.1371/journal.pcbi.1010706)
Supplement: Attachment — Submitted filename: Response_to_review.pdf [file pcbi.1010706.s009.pdf]

Reviewer 1

This is a very nice paper, providing an extensive examination of information theory and pattern separation in the dentate gyrus. This is a complex study and is complicated by combining two complex and awkwardly discussed subjects in neural computation: information theory and pattern separation. However, I believe this paper does an exceptional job at using the former to help constrain the discussions of the latter, and as such, I think this is positioned to be an incredibly important study.

The main strength of this paper is its central premise – examinations in pattern separation in the dentate gyrus are poorly constructed because they fail to account for the information encoded by the neurons. This is, in large part, because the notion of pattern separation is often conflated with sparsity. One can get pattern separation in several different ways – one can get it by lowering firing rates in a randomly activated network – this was the premise of early DG studies like O'Reilly and McClelland [which the authors should probably cite] and Treves and Rolls; and one can get it from maintaining overall levels of activation yet actively selecting different neurons to be active, which is the premise of a lot [but not all] of studies modelling adult neurogenesis. Summarized briefly, "pattern separation" alone is an under-constrained concept and requires an accompanying notion of "information" to evaluate properly.

We would like to thank the Reviewer for their appraisal of our work and many helpful comments, which we have addressed individually below. We have added a citation to the 1994 O'Reilly & McClelland paper when discussing how sparsity contributes to classical measures of pattern separation.

The paper discusses several formal measures of pattern separation and of information theory and relates them in a way which goes a long way towards achieving that goal. I have several comments that I think can make the paper stronger and more impactful.
• Provide a more direct and thorough historical context for the paper.
o This paper goes through a lot of references, particularly in recent years, that provide a strong grounding of the computational neuroscience relevant to this paper.

o The paper needs a bit more rigorous discussion of how in vivo physiology [and accompanying analyses and interpretations] has biased the perception of pattern separation in the dentate gyrus.

This is a very complicated field, made in part more complicated by the fact that the community which typically studies and quantifies in vivo activity in the dentate gyrus tends to not pay a lot of attention to neurogenesis [but see Rangel et al., Nature Communications 2014 and Rangel et al., Frontiers in

Neurogenesis 2013 and Danielson et al., Neuron 2016] and uses rather old notions of pattern separation; while the computational literature in the field in recent decades has increasingly paid attention to the heterogeneity of the network offered by neurogenesis.

Fair or not, in vivo physiology drives the mainstream theoretical perspectives in hippocampus field. While the results described here are best studied and demonstrated in computational models [for which there is total control], the biggest impact of this approach will be taking these techniques into in vivo physiology data.

We would like to thank the Reviewer for these insightful comments. We have added acknowledgements in the Introduction and Abstract that the gap between in vivo *studies and computational work has contributed to the continued usage of potentially flawed metrics of pattern separation and neglection of neurogenesis, and further highlighted in the Discussion that our new measures are highly suitable for experimental, as well as theoretical, studies. We have also added some of the references mentioned by the Reviewer.*

o More historical context around neurogenesis.

This paper does a great job of pointing to recent studies of neurogenesis, but I think it is fair to say the debate about information theory and pattern separation is fundamental to the neurogenesis field, going back to some of the earliest modeling and theory papers [most of which are not cited here]. The neurogenesis field has wrestled with the conflict that this paper addresses for longer than the authors may realize. In particular, Neuron published two papers back-to-back (a Point / Counterpoint) in 2011 [Aimone, Deng, Gage; Neuron 2011, Sahay, Wilson, Hen; Neuron 2011] that, in a sense, directly debate the question that is being addressed by this study. Summarized a decade later, the distinction is that the Sahay paper argued that new neurons role is to increase pattern separation through increasing sparsity by virtue of increasing feedback inhibition, whereas the Aimone paper argued that new neurons role is to increase information content [referred to as "memory resolution"] in the hippocampus.

We agree that neurogenesis is fundamentally linked to the problems addressed in this paper and thank the reviewer for this suggestion. We have added an acknowledgement of Aimone at al's concept of 'memory resolution' and how this relates to our new measures in the Introduction, and a brief summary of the debate over neurogenesis, including the Sahay et al paper, in our Discussion.

• The paper combines single cell and population level pattern separation and information theory, which is awkward.
o As the authors note, pattern separation is a circuit concept, though can in certain circumstances be

studied from a single neuron perspective. Likewise, information theory is best described for single neurons yet is challenging [but important] to scale up to circuits. I think this paper does a nice job of trying to bridge both scales, but it remains a bit awkward [to me] as I don't have a great intuition of what the single neuron simulations (at moderate levels of complexity) mean. The information theoretic measures make sense, but I don't really follow why one would even look at pattern separation here. That may be the point being argued [for single neurons, one should focus on information, not pattern separation], but I think the paper should be more explicit about this.

We agree with the Reviewer that pattern separation primarily occurs at the circuit level. The single neuron simulations are presented as a direct parallel to recent experimental studies on pattern separation by Madar, Ewell, and Jones, which directly accessed the inputs and outputs of a single neuron (although the single neurons were not isolated form their circuits, as we now make clearer). The idea here is that single neurons contribute to pattern separation independently of network effects by mapping similar inputs to less similar outputs; if similar inputs are presented one after the other, then the dissimilarity in outputs can be quantified and a measure of single neuron pattern separation recorded. This is a proxy for a population of independent neurons receiving similar patterns simultaneously. We have clarified this point in the Results.

o While the authors cite several papers discussing pattern separation of individual neurons, there are other papers as well that have explored how differences of individual neurons can be involved in channel decorrelation [eg., Mishra and Narayanan, Hippocampus 2019, and other papers by those authors].

We agree that heterogeneity in a neural population is likely to contribute strongly to pattern separation performance, and now acknowledge this explicitly when discussing our network simulations, which already include heterogeneities between neurons.

• The figures, overall, are informative and thorough but should be worked on.
o Somewhat obvious, but this paper has a lot of data presented in figures. I count something like 75 panels in just the main text alone (not counting the more populated supplemental figures). Adding to this, each figure has several sets of results plotted on them, and each figure has to be evaluated somewhat independently as the format of data and axes vary considerably across the paper.
o I would strongly suggest that the authors step back and ask for each figure what is the dominant message that is trying to be communicated, and ensure that *that* is what is best shown in the figure.
 For instance, Figure 1 has a pretty fundamental point – if you filter data to sparsify it, you increase pattern separation but decrease information content. This result is rather consistent regardless of which filtering approach is used, which separation measure is used, and which information measure is used.

The paper is not about filtering approaches, however, so it likely is sufficient to just show the random filter data [or whichever you choose] and only show the top left subpanel of each panel, and simply state that similar results are seen in the other filters. That will drive home the main takeaway message, and also allow the reader to better appreciate the differences between the separation and information metrics.
o I would also caution the authors from using figures that cannot be easily interpreted. For instance, the input and output panels in figure 3 are, I presume, intended to show how the neuron provides distinct outputs to different inputs. While the output figures (in particular) are attractive, they are too small to really interpret anything from.

We have considered the Reviewer's suggestions and simplified the presentation of several figures in the main text. For Figures 1 and 2, we have simplified the panels to show only the uniform random filter. For Figure 3, we have moved the simulated voltage traces to the corresponding supplementary figure and replaced them with the panels requested by Reviewer 2 showing the components of our new measures independently.

• The network simulations are really small.
o Central to most of the theories about the dentate gyrus is its size relative to other regions. It has a lot of neurons. This is also somewhat central to the importance of neurogenesis, whereby new neurons represent a very small but correspondingly hyperactive set of neurons in the network.
o While a mouse DG is 300,000 neurons; this paper simulates 50 granule cells, with a half dozen or so interneurons. This is a really small number. Yes, there will be computational limitations due to the high fidelity of the neuron simulations; but there is no reason to only simulate populations of neurons at that level of fidelity. Further, it is 2022 and computing resources being what they are, the authors should be able to go beyond a few dozen neurons.
 The main problem with networks of this size is the diversity of inputs. If multiple neurons in a network share a common set of inputs, they will end up with correlations that will propagate through all of these measures being considered. The degree of correlations of inputs is highly related to the scale of the inputs population, number of synapses, and number of neurons in the network.
 A second problem with networks of this size is that adult neurogenesis is very connected to scaling – both in terms of the number of synapses arriving on immature neurons and the breadth of the sampling the new neurons take.
 Stated differently, if the authors were to model 1000 GCs sampling 100 EC neurons (100,000 sampling 10,000) at a very low level of detail [and some of the papers the authors cite do just that], the dynamics will obviously be a lot less informative, but most of the pattern separation and information theoretic metrics should apply just the same. It wouldn't surprise me if the authors see rather different behaviour

because the separation and information content is kind of baked into the sparse and dispersed projections.

We have expanded our network simulations to include larger numbers of simplified granule cell models (see the new Figures 7 and S6) with diverse input properties. We find that our measures remain informative when applied to such large networks. We agree that there is much further work to be done on the role of neurogenesis in pattern separation, but our main intent in this paper is to introduce and justify our new measures. We hope to apply them to such questions in the immediate future, and our accompanying pattern separation toolbox will allow other researchers to do the same.

Reviewer 2

This manuscript makes the case for including considerations about information transmission when studying pattern separation in the context of episodic memory and dentate gyrus processing. A systematic analysis of different types of similarity metrics confirms and extends past results that sparsity and information-destroying unreliability are sufficient to perform pattern separation. The authors then introduce 3 new metrics (and ways to estimate them with sparse data), based on common information-theory measures (mutual information, transfer information or redundancy). These metrics are designed with the goal of detecting pattern separation that preserves information. The use of these new metrics is displayed in different contexts: a biophysically realistic single granule cell model receiving cortical inputs, and a small model of the dentate gyrus network with normal or pathological parameters. The analysis of the single GC simulations with the new metrics 1) confirms that different facilitation and depression parameters yields different pattern separation levels, 2) identifies short-term dynamics that maximize information transmission, and 3) shows that conductance heterogeneity does not impact pattern separation, confirming the degeneracy of these parameters. The analysis of the network simulations confirms that different cell-types can impact separation and information transmission differently, and suggests that the pathological increase of input strength and intrinsic excitability observed in epilepsy can both lead to information loss and decreased separation, which is new and interesting but may be confounded by the way the new metrics are designed.

I think the use of information-theory approaches to better understand dentate computations and pattern separation in general is a valuable endeavour. The methodology is rigorous, and the presentation of the results is neat, relatively clear and put in the context of the relevant literature, but the interpretation of the results needs to be fleshed out for the reader to learn how to interpret the

new metrics and to better understand the significance of the proposed methods. My main concern is with the design of the new metrics. In particular: 1) I am not convinced that the sparsity-weighted mutual information is measuring pattern separation, 2) I think combining information transmission with similarity measures creates interactions that are difficult to interpret and that both aspects of a networks' computation are best analyzed separately.

We would like to thank the Reviewer for their appraisal of our work, the in-depth review, and many helpful comments, which we have addressed individually below.

1) We agree that the sparsity-weighted mutual information Υ_M does not necessarily *measure pattern separation, but argue that it is an intuitive measure that will reflect pattern separation in the vast majority of cases and is easier to compute (and explain) than the relative redundancy reduction Υ_R. This is because a sparser code that retains information is likely to be using an encoding of higher dimensionality (one of the ideas behind the compressed sensing papers we discuss), and this higher dimensionality causes patterns to separate, as argued in Cayco-Gajic & Silver (2019).We now make this point more explicitly when introducing $\Upsilon_{M.}$*

2) We also agree that it is often informative to consider the different aspects of pattern separation as a computational function (reliable information transmission and reduction in similarity) separately (see below), but would argue that there is considerable value in a single measure that can be used to answer the question 'Does this feature improve or hinder information-preserving (ie non-destructive) pattern separation?'. The central argument of our manuscript is that pattern separation should not be evaluated without consideration of the information throughput, without this the very idea of there being 'patterns' to separate at all is lost. Consider the extreme case of a black-box neural circuit that takes any input and produces a completely random output that is entirely independent of the inputs. What could the function of such a circuit be, no matter how well separated the outputs are? It would not have a role in the indexing theory of Teyler & DiScenna, which we discuss further below, as no activity patterns could be reliably indexed. Nor could it be used either to train or recall patterns in a classical completion circuit. We argue that such a circuit would not be a 'pattern separator', but a 'pattern destroyer', and this is the reason for our decision to incorporate feedforward mutual information (or transfer entropy) directly into our measures.

To show how the two components of our measure contribute to the final values, we have expanded Figures 3 and 6 (formerly 5) to show the sparsity, pure mutual information, and pure redundancy reduction measures in some cases (panels 3c & 6f), and have further clarified the motivation for and interpretation of our measures in the Discussion. The new Figure 5 also illustrates how the combined measure Υ_R can be predictive of pattern completion accuracy in a toy model of an auto-associative network.

Comments:
1) Line 33-77: The 2nd paragraph of the introduction is both 1) a presentation of past strategies to measure pattern separation and 2) a motivation for the study by showing problems in the current way pattern separation is measured. I think conflating the 2 points is confusing. To make things clearer, I suggest separating the review of past similarity metrics (they all have their pros and cons and assumptions, including different dependencies to sparsity and firing rates), from the motivation for the need to better control for information loss when studying pattern separation.

We have separated this paragraph in this way, as well as dividing the new second paragraph to focus on the parameter-dependence and information-loss issues separately.

1a) One potential problem with similarity metrics is their dependency over firing rates or network sparsity. This is touched upon diffusely in the manuscript (line 53-57, line 98-100, line 123-4, line 197, line 449) and some of the confusion might be reduced if it was addressed separately from other issues or motivations. This point seems particularly relevant to results in Figure 1 (paragraph starting line 118) and to explaining the "sparsity-weight" used in the design of the new information-theory metrics. I also think it should be noted that most past studies cited on the role of sparsity in pattern separation (e.g. line 123) have considered a binary population code, and thus network sparsity rather than spiking sparsity as is the case in this study. Cutts and Eglen 2014 (not cited) and Madar et al 2019b (cited), seem most relevant to the present study, as they both have interesting discussions on the effect of firing rates on similarity metrics for spiketrains.

We agree that dependency on firing rates is another potential problem of similarity metrics, but that is a separate issue and not obviously linked to the sparsity component of Υ_M *as it specifically biases binarised metrics. We have added a more explicit acknowledgement of sparsity as a distinct issue in the Introduction and cited both the Cutts & Eglen (2014) and de la Rocha et al (2007) papers that discuss this issue.*

1b) Line 58: the issue of bin size choice noted for classical similarity metrics remains for the information-theory based metric presented here: these new metrics are not parameter-free and choices must be made concerning the type of neural code investigated and the word and/or letter length.

Whilst different choices of neural code and parameters will typically lead to different measures of information content, it is possible to pick these in a principled way to maximise the measured information as we do here. It is not all clear that similar processes can meaningfully (or even practically in the case of Figure 1b) be used to pick bin sizes that maximise, for example, orthogonality. We have added a sentence to clarify this.

1c) Line 51, authors point to inconsistent results between studies, but most of these apparent inconsistent claims are first due to differences in what patterns of neuronal activity were considered, so they are not really inconsistencies. Many studies indeed claim to measure "pattern separation" but actually refer to a broad class of computations. This makes the literature confusing, because findings are really about different neural codes and computations. In this paragraph, authors have grouped and compared studies that considered either ensemble of binary neurons or spike trains in single neurons, or different types of spatiotemporal patterns. This unaddressed diversity makes the authors argument somewhat difficult to follow and potentially misleading, as the pros and cons of each metric won't be the same or mean the same thing for different types of activity patterns.

We agree that many of the studies we cite use different neural models, codes, and computations. We have added a clarification of this point in this paragraph, but also argue that the inconsistent definitions and conceptions of pattern separation in the literature rather support our argument that it is important to search for better measures.

1d) Line 65, it is said that Vineyard 2016 and Madar 2019 explicitly note that the fact that information-destructing noise can lead to high pattern separation levels is a limitation of standard metrics. That is not my reading of the literature: the metrics are not intrinsically at fault and those papers do not make that claim. Pattern separation is not defined in terms of information, so it may (or may not) be performed by purely random processes, and the papers cited note that trade-off between pattern separation and reliability of information transmission. Measuring pattern separation and reliability are two different questions: one is quantifying the degree of separation and the other is about the mechanism underlying the separation (random information-destructive process or not). I agree that both are important to test the role of pattern separation in episodic memory, but the concepts are separate.

We agree that the existing metrics are not at fault, rather that they are intended to measure something we consider to be only part of pattern separation as a useful computational function. Our central argument is that pattern separation, as opposed to what we call pattern destruction, depends on both the transmission of information and a decreased similarity between inputs. We argue that these concepts cannot be usefully separated in most cases. We have changed 'note this as a limitation of standard pattern separation measures' to 'note that standard pattern separation measures do not consider destruction of information' here to better reflect the intent of the literature.

2) The authors' premise and justification for their new metrics is that information destruction is undesirable for pattern completion and episodic memory to work properly.

2a) I do not think that this premise is obviously true. It depends on when pattern separation is performed. Pattern separation is theorized to happen during encoding and pattern completion during recall. When those two computations are separated in time, using an unreliable process to pattern separate during encoding does not then necessarily preclude recall in CA3 at a later time point: DG might not perform pattern separation during recall, or might be decoupled from CA3 during that phase, leaving only the EC input to CA3 to trigger pattern completion (cf Treves and Rolls 1992, O'Reilly and McClelland 1994).

We agree that unreliable processes are key to the function of the hippocampus, but would argue that separation in time does not help with the loss of information in the dentate gyrus. As we are considering granule cell, rather than CA3 activity, there must still be a relationship between the input and the output for the episodic memory to form at all. We take the Reviewer's point into account and explicitly consider the training/recall separation in the Introduction and mention it again when discussing our results around the new Figure 5.

2b) Moreover, noise is not necessarily a bad thing when it comes to pattern separation, and can be a useful feature rather than a bug, for instance promoting robustness or efficiency in population codes (see Wolpert 2008, Hunsberger and Eliasmith 2014 or Washke and Garrett 2021) and even information transfer in some cases (e.g. Goldberg 2004, a paper that the authors cite). As pointed out in Madar et al. 2019a, in the case of episodic memory the unreliability can be meaningful and carry information about the time of the episode being stored. The pattern separation process could even potentially fully destroy all the information content from the cortex and CA3 would just store a randomly-generated index, which corresponds to the original index theory of episodic memory (Teyler and Rudy 2007, Legendy 2017).

We agree that noise is not necessarily a bad thing for information transmission and certainly not for pattern separation. However, we do not think that the index theory of episodic memory is an argument against our metrics as they would still be able to identify the effective generation of a reliable index through the mutual information component and so would hold up far better than the classical measures we consider. We have added a brief discussion of this idea, and the related points of the Reviewer, in the Discussion.

2c) Line 34-36 "When considered as part of a system preceding pattern completion, good pattern separation can be simply taken as any procedure that reduces noise and enhances the storage capacity of the downstream network": As hinted above, the assumption that good pattern separation should "reduce noise" is debatable (especially given the wide array of definitions that the term "noise" can take. However, the fact that the form of pattern separation we are most interested in

the context of DG and episodic memory are the ones that help increase storage capacity in CA3 is true and at the center of the authors argument and motivation for designing new pattern separation metrics. So, they should show that their methodology is able to distinguish between "good" and "bad" pattern separation, by showing that high pattern separation levels as assessed by their metric necessarily lead to improved storage capacity without catastrophic interferences.

Following the suggestions of the Reviewer, we have added an additional section to the Results ('Information theoretic measures can predict pattern completion', the new Figure 5) showing how noise can both enhance and impair the recall accuracy of an auto-associative network, and how this recall accuracy is predicted by the relative redundancy reduction.

2d) Similarly, it would strengthen their argument if they could show that information-destructive pattern separation is necessarily bad for subsequent pattern completion. I guess this brings us back to the preceding point, as it's not clear that a separating process that completely destroys the information contained in the inputs would impair storage if the role of the dentate is to generate a random index that informs on the time of the episode (Legendy 2017). DG is not the only source of input to CA3 and is often hypothesized to not be particularly important to trigger pattern completion during the recall phase (Treves and Rolls 1992, Hainmueller and Bartos 2020).

As mentioned above, we have added a new Results section (around the new Figure 5) that goes some way to addressing this point. Whilst it does not show that information destruction is necessarily *bad for pattern completion, it does show that destruction of the information necessary for pattern completion is bad for pattern completion. This is consistent with our assumptions discussed elsewhere that the dentate gyrus should maintain information throughput.*

3) All that said, I think using information-theoretic approaches to quantify pattern separation makes a lot of sense. Shannon's entropy is a natural concept to apply to assess the variability in a group of activity patterns, and whether this variability is reduced in the output.
Line 189: Measuring redundancy in particular does appear as a useful and genuine similarity metric. Moreover, figuring out whether the redundancy in inputs has been removed in the output is an important hypothesis that has been challenging to test. The combination of mutual information measure with redundancy could help. Can such measure identify which parts of the inputs are redundant and check whether the output ensemble retained the non-redundant spikes?

This is a complex question to answer in general, as which spikes are redundant will depend on which other spikes are removed, which itself might shift the optimal encoding. If one has a strong biological or theoretical reason to

consider a specific spiking code, it would be possible to check this directly and we now acknowledge this as a useful possibility in the Discussion.

4) Input correlations: could you note in figure 1 that these correspond to the phase-locking strength (as opposed to Pearson's correlation, or input similarity measured through each plotted metric)? Showing the pattern separation levels for different input correlations is important and interesting, however I see multiple issues or sources of confusion here.

We would like to thank the Reviewer for pointing this out, our usage of 'correlation' for the strength of the relationships between different parts of the input over different patterns was unnecessarily ambiguous here. We have clarified this throughout.

4a) phase-modulation strength is yet another "non-classical" similarity metric used in this paper, which could have been used to measure the input and output similarities.

We agree that phase-modulation strength could be a useful similarity metric in some circumstances, but feel that including it here would make our manuscript less readable for limited benefits as it lacks the novel features of Υ_M *and* Υ_R*. We have therefore decided not to include it in our revision.*

4b) One generally wants to know how pattern separation levels differ for different input similarity levels measured the same way that output similarity was measured. It would be useful to show that directly, or to show how phase-locking strength relates to the other metrics used.

We agree that different levels of input similarity are material to the level of pattern separation, but feel that our presentation of different phase-locking strengths show how our new metrics handle this. To address this point, we now make this clearer in the revised manuscript. In particular, Υ_M *is relatively unaffected by input similarity but* Υ_R *often depends on the input redundancy which reflects the input similarity. In terms of classical measures, Figure S1 shows pattern separation for low, medium, and high levels of input similarity (over different correlation structures). Figure S3 does this for the information theoretic measures.*

4c) relatedly, since the plotted pattern separation levels are averages over all pairs of spiketrains in an ensemble, it would be important to report the range of input and output similarities in the ensemble. Are they always well constrained around the mean for all metrics and conditions?

In our simulations, the range is well constrained, but this is also a feature of the length of the ensemble, with shorter ensembles being more variable between trials. We have added a sentence to clarify this.

5) Methodology to measure pattern separation:

5a) The type of activity pattern considered should be clarified as early as possible in the main text and/or figure 1. If I understand correctly, the method section makes this explicit, but it would help the reader to say in the result section that only temporal patterns are considered (i.e pairs of non-simultaneous spiketrains are compared, and the input similarity is the average over all spiketrains of the input ensemble). This is important because it helps comparing to other studies that have or have not considered the same type of patterns (e.g the Madar studies, as opposed to, say, Yim and Wolfart 2015 which considered spatiotemporal patterns). In particular, for the Hamming distance, this metric is usually used on population patterns rather than spiketrains. So, the results in Figure 1 are not directly comparable to the Myers studies or the Poirazi studies.

We thank the Reviewer for pointing this out and have now clarified the type of activity pattern and the differences to the literature before Figure 1.

5b) Pattern separation levels are not measured as differences (Sinput – Soutput) but as ratios (Sinput/Soutput). This different from Madar et al. 2019 (to which the results are compared to) and to most past studies, and should be discussed. Could the authors explicit their rationale for that choice? I feel like Sin/Sout is not as easy to interpret as the difference. Especially if the similarity metric is not bounded between 0 and 1 like the Wasserstein metric. Details on the range of values for each reported metric and on how to interpret them would be useful.

We chose to use ratios to normalise the pattern separation as the output similarity relative to the input similarity in order to minimise the input similarity as an additional source of pattern separation variability (as in Madar et al) and keep the measures as consistent as possible. We agree that this is one of several possible choices and now acknowledge this more clearly.

5c) More details should be provided on the rationale behind the design choices for each sparsity-weighted information-theory metrics, as well as more details on how to interpret the values. Overall, I do not understand how sparsity, mutual information, transfer entropy and any combination of these can be considered measures of pattern separation, as these do not measure the similarity between spike-trains.

We have now clarified our rationale for these choices and discuss different perspectives on pattern separation in the Discussion.

 Line 198: What is “differentiation” here? A synonym for separation? How is sparsity a similarity or separation metric?

We have replaced ‘differentiation’ with ‘pattern separation’.

 equation 1 shows that the ‘sparsity weight’; is the difference of number of spikes normalized to the input number of spikes. That means it’d be negative if there are more spikes in the output: is that desirable?

The Reviewer is correct, the sparsity weight would be negative if the output contained more spikes than the input. We believe that this is a desirable property as it would indicate a denser, potentially lower dimensional representation (see above), of the inputs and hence would likely be counterproductive for separating patterns. We have added an acknowledgement of this possibility when introducing the metric.

 I also wonder why normalizing to the input sparsity (as opposed to an average of input and outputs, or their sum, or something else, if the goal is to make the metric less dependent on pairwise firing rates).

The reason for normalising the sparsity to the input is to get a measure of how sparse the output is relative to the input: if the input code is already quite sparse there will be relatively less space to remove spikes further. The metrics are not specifically chosen to minimise dependence on paired firing rates as long as the total number of spikes are minimised. We have now clarified this when we introduce the sparsity-weight.

 I don’t see how this sparsity-weight is a similarity metric or how it assesses pattern separation. As I understand it, sparsity is a feature of a spike-train, not a similarity measure. But one could design a similarity metric that would evaluate the dispersion (or entropy) of sparsity across multiple spike-trains, as in Madar 2019b. That is not what is done here, as far as I can tell.

We agree with the Reviewer that sparsity-weight on its own is not a measure of pattern separation and we now make this more explicit. When combined with mutual information, however, we argue and show that it is indeed a useful measure of pattern separation as it implies a higher-dimensional code with more space in which to separate patterns. Our redundancy-reduction measure is a direct measure of the informational independence of different spike trains in an ensemble and usually correlates closely with the sparsity.

- Line 200 “the simple sparsity measure is preferred as it makes no assumptions on the pattern completion circuit: not sure what is meant here. What assumptions do other measures make that sparsity doesn’t?

The other, classical, metrics make implicit assumptions about what features of a spike ensemble should be separated (orthogonality, scale, correlation, or some distance), as well as having the parameter dependence discussed above. We now clarify this.

- Line 200 “requires no discretisation of spike times”: if sparsity is simply the number of spikes, then there is a discretization choice in the length of the spike- train considered. The choice of spike-train length is as arbitrary as a bin size. Madar et al. 2019b consider such a measure as the “occupancy” feature of a spike train for a time bin as long as the total spike-train duration.

We agree that the length of the spike train can be arbitrary, but that is equally true for the bin-based measures, which then also have to use a bin size. Further, in some circumstances it is useful to define an information rate (in bits/s) that would avoid this complication and which we now mention in the Discussion.

5d) Line 204-218: I am not convinced by the idea of multiplying similarity (or sparsity) measures with information transmission measures. Authors argue that they want to measure the “balance” between the two, but combining them makes the interpretation more difficult, as there are possible interactions. For instance, a situation with low redundancy reduction but high mutual info would give a similar value than a high redundancy reduction and low mutual info scenario, wouldn’t it? Providing more details on such cases would help the reader interpret the results throughout the manuscript. I personally think it’s easier to interpret when separation and information transmission are not combined. The redundancy difference can be used as a very useful pattern separation metric. The mutual information can be evaluated separately.

Yes, a circuit that produced outputs with high sparsity and low mutual information might have similar pattern separation performance to one that produced outputs with low sparsity and high mutual information under our metrics. Typically there is a peak of circuit performance with intermediate sparsity and mutual information (Figs 3b & c). We clarify this point when introducing the metric and further discuss many of our results in terms of the two effects separately.

5e) The neural codes assumed for information-theory metrics are not precisely defined. What was the word length, letter length, and other relevant parameters? These parameters should be somewhat justified. Is this where the authors chose the strategy of maximizing information, as suggested line 224?

(I don't see why this particularly more principled than using biologically relevant timescales or checking multiple timescales, as detailed below in point 6. We don't know that the code is optimal).

The codes are taken as a maximum over different word lengths and bin sizes. We also perform this maximisation for a variety of codes and select the one with the highest information content. The goal is to identify the maximum amount of information that could possibly be contained in a given signal and so correctly penalise the absolute loss of information, however measured. While this is practically hard to do, we believe that it is fundamentally the right approach for this reason. We now clarify how and why this is done in the text when discussing Figure 2.

5f) It would be interesting, and useful for interpretations, to explicitly show how the new metrics relate to classic pattern separation metrics (for now, we have Fig 3B and Fig S4 to compare). For example, Fig 3B (line 249-52) suggest that there's a correlation between the redundancy reduction measure and more traditional correlation measures (e.g. phase-locking strength, which would be picked up by Pearson's R too), which is expected if it's really about redundancy. And there's no correlation with the mutual info metric, which makes sense if it's not based on similarity measures.

There is not a consistent relationship between our new measures and the classical measures we consider (see for example the central column of Figure S4 for correlations). This is because the new measures balance the two aspects of pattern separation we consider important, and the classical measures do not consider information transmission. This is apparent in Figures 1 & 2, Figures 3 and S4, the final panels of the new Figure 5, and the new Figure 7. Our new measures typically take low values for both low and high values of classical measures.

6) Line 156-157: This is debatable. The discretization choice is not necessarily arbitrary, some timescales are relevant for biological reasons: 10-20ms corresponds to membrane time constants, over which inputs can be integrated. 100-250ms corresponds to the theta rhythm which modulates entorhinal and hippocampal spike-trains. Multiple timescales are likely relevant and carry different meaning, so I think it is wise to check several of them. Given that the relationship between bin size and pattern separation levels is continuous, it's ok to check only a few. The bin size parameter is not a trivial issue but, in my view, it is not an intrinsic disadvantage of binned metrics compared to metrics based on raw spike times like the Wasserstein distance: it depends on how spike-trains are read by downstream targets. Raw spike times may be irrelevant in some cases. More likely, multiple timescales are relevant.

Moreover, as said in point 1b) above, information-theory metrics presented in this manuscript suffer similar issues of discretization choice. This is acknowledged in the methods (line 458) where it is mentioned that "the coding strategy and discretization should in principle be chosen to maximize

information”. I don’t see why this is particularly principled, and why the same could not be done for classical metrics (i.e. finding the timescale that maximizes similarity).

We agree that specific timescales may have biological meaning, and now acknowledge this more clearly in the text. The information-theoretic measures are able to choose a code and bin size that maximises information content for the principled reasons described above (response to 5e). We did not find that such an approach is generally possible for many classical metrics (see our Figure 1b) and so could not generally be applied. We do consider this to be a disadvantage of classical binned methods, despite the ability to look at several different timescales, although it is less important than the issue of information loss.

8) Presentation of the results: In Fig 1 and 2, where multiple conditions, neural codes and similarity metrics are represented, it is hard to map the colour shade to the legend. The graphs are dense in information, so increasing contrast between colors/symbols or separating plots would help.

We would like to thank the reviewer for this suggestion. This point is similar to a point by Reviewer 1 and we have followed their suggestion to simplify the presentation of these results in the main paper. As the effects we describe are consistent across the different filtering methods, we have reduced the number of panels by focussing on the uniform random filter in Figures 1 and 2. We hope that this has made the figures much less cluttered and easier to read. The corresponding supplementary figures keep all of the different filters.

9) Interpretation of the results: Throughout the manuscript, the interpretation of the results could be fleshed out a lot more, making more explicit what the results reveal about the meaning of each metric or about the modeled system (singe GC or DG network). Some examples:
 on line 163, what do the non-monotonic behaviours for the decorrelation and Wasserstein measures mean? Why do they occur?

The non-monotonic behaviours from the Wasserstein measure with a refractory filter and the decorrelation measure with a competitive filter may have specific meanings as these filters interact with these measures in different ways. We are not able to give an intuitive answer to why these specific interactions produce non-monotoniciy and feel that a deeper exploration would distract from the main narrative of the manuscript.

 Line 222 “In all cases there is a single peak at an intermediate value of the thinning filters.”: Why? What does that mean?

We have clarified that there is a single peak in each measure. The reason for this intermediate peak comes from the opposition between the two components of our measures.

- What do the peak of the curves in Fig 3B mean?

The peaks indicate parameter values which give the best pattern separation under our metrics, where the decrease in information transmission balances the decrease in spike train similarity. We now emphasise this further.

- The meaningfulness of Fig 3 and 5 results is not clear. More explicit interpretation would help the reader.

We have followed the suggestion of the Reviewer and expanded our discussion of these results.

10) Line 230: Why is gamma_M concluded to be the most useful pattern separation metric? Since I don't understand how gamma_T and gamma_M can be considered pattern separation metrics, that conclusion is far from obvious. The argument for them as pattern separation metrics and for how the authors rank the usefulness of each metric needs to be explicit. Similarly, in the discussion (line 343) it is claimed that "We have demonstrated our new techniques on single cell models and shown that they allow more useful conclusions to be drawn". I disagree with the argument presented on line 329-39 (for reasons detailed above) and don't see how gamma_R for instance is necessarily more useful than other classical measures. They may be complementary, as they make different assumptions on the neural code and measure different things. The argument for preferring the use of gamma_R (or gamma_M) over any other metric needs to be bolstered or the claim modified.

We believe that Υ_M and Υ_R are the best pattern separation metrics as they balance the cost of lost information with the decrease in input similarity. We have demonstrated both in abstract models (Figures 1, 2, & 5) and more realistic neuron and network models (Figures 3, 6, & 7) that these processes are typically opposed to each other and must be considered jointly to avoid confusing pattern separation with pattern destruction. We have expanded our reasoning here.

11) Figure 3:
a) 3A + line 241: what does each group of 5 input patterns represent? 5 different patterns at the same synapse, with a certain correlation structure? I imagine there are 4 groups for the 4 strong yellow synapses. Please clarify how these inputs were made and what synapse they are stimulating when.

The 5 inputs are presented sequentially to all four synapses at once and represent different components of the pattern. This is now clarified.

b) 3B: what was the neural code considered?

The neural codes for the input and output are chosen to maximise the informational measures to cover the best-case scenario of information content. We have clarified this in the caption.

c) "Spike traces are two minutes long and consist of phase-locked inputs with a phase rate of 0:6Hz and a spiking rate of 5Hz." Why those parameter choices? Do EC inputs show such a slow-frequency modulation and firing rates? From Desmukh and Knierim (2010) and Pernia- Andrade and Jonas 2014, there's a clear theta-modulation of EC inputs, seemingly mostly due to MEC inputs. Not sure about slow oscillations. LEC inputs don't seem to show much phase modulations, based on Desmukh and Knierim 2010.

These choices were taken to keep inputs consistent between Figures 1, 2, 3, 4, & 6 and make results as comparable as possible. The phase locking was taken to allow for spatiotemporal correlations (although we also consider spatial and temporal correlations separately in Figures 1, S1, & S2). We now explain this here and have included firing patterns based on grid-cell like firing patterns in the new Figure 7.

12) Figure 4: It is useful to show how long the trains must be. An other graph, showing how much data is needed (how many spiketrains, how many repetitions, etc) to be able to compute accurately the exact values would help experimentalist design their experiments so that they can use those methods.

We agree that it would be very useful to be able to plan the necessary length of experiments for our measures to be accurately applied. There are, however, a large number of other factors that influence this such as the type of input spike patterns, the experimenters control of them, the strength of connections and 'noise', and the excitability of target cells. We were not able to cover all possibilities and would not like to give potentially inaccurate rules-of-thumb here. This question would best be answered in a specific case, ideally with some preliminary data, and we now highlight how such an approach could be used here.

13) Line 285, Figure 4: How many spiketrains are considered in the input and output groups? Does the number of spiketrains being compared impact the accuracy of the exact MI calculation or the KL estimator? What about the correlation structure of the spiketrain ensemble: does it affect MI exact and estimated values?

As before, 5 spike trains are considered in each group and we now clarify this in the results. We were unable to identify systematic relationships between the accuracy of the estimators and the number of spiketrains, though in principle larger ensembles may be more informative.

14) “Pattern separation in a network” Line 289: Here, despite considering cells integrated in a network, pat sep is still evaluated at the single-cell level, considering only temporal patterns, correct? Or are spatiotemporal patterns (i.e. population of simultaneous spiketrains) considered?

This is incorrect, the collection of spike encodings considered includes spatiotemporal patterns and we have now clarified this.

15) Figure 5C-E: as for Figure 3, please clarify the neural code assumed and why this code was chosen.

The informational quantities are maximised over the set of codes described in Methods. We now clarify this.

16) Fig 5B: So, each of the five groups of GCs receives 1 of the input rasters displayed? The same raster is activating multiple synapses on each GC? Please clarify where and when input patterns are delivered.

We have moved the description of the inputs from the Methods to the Results.

17) Fig 5C: As pointed out above in comment #4, it’s confusing to display different measures (one being correlation, the other redundancy reduction) for inputs and outputs. Why not just show redundancy for both inputs and outputs, on one hand, and show how redundancy reduction relates to decorrelation (or phase-modulation strength, in your case) on the other?

Whilst we do not believe that the combined measures are confusing, we now plot the individual components in panel F and discuss them.

18) Line 302-5, Figure 5D: Since scaling the synapses strength up is done, why not show the effect of scaling down as well (using Jungenitz 2018 to constrain the parameter range), since you hypothesize its importance for pattern separation levels in abGCs.

The goal of this panel is to demonstrate clearly that the strengthening synapses in the way that is seen in epilepsy is detrimental to pattern separation. We feel that down-scaling synapses here (in the pathological case) would be unnecessarily confusing, but consider both upscaling and downscaling input rates and recurrent synaptic strength in a larger network in the new Figure 7. This is also an interesting question when applied to adult-born

granule cells, which are less integrated into their circuits and can receive weaker inputs. We look forward to studying this in more detail in future.

19) Fig 5E and S5B: Both panels look surprisingly similar: Why? If I try to interpret the results (which the authors should expand on), it suggests that a pathological increase in intrinsic excitability, or in input strength, or both, leads to information loss (as assessed by the sparsity- weighted mutual info) and a decrease in pattern separation (as assessed by the redundancy reduction). This would be unexpected, since information-destructive unreliability generally improves better pattern separation (as shown in Fig 1). But, as pointed before (comment #5d), the similarity between 5E and S5B may simply result from the fact that the redundancy reduction metric is combined with the mutual information. So maybe the information loss is just driving the result in both figures here, not the redundancy reduction. This is why keeping separate the measure of pattern separation and the measure of information transmission is so important for interpretability. If the conclusion that indeed both pattern separation and information transmission are reduced in epilepsy, then more analysis to understand how epileptic alterations impair both aspects of dentate computations would be very interesting.

The Reviewer is correct that the similarity between (the now) Figures 6E and S5B (as well as the left and right columns of Figure 3B) are driven by i) the inclusion of mutual information in both the sparsity-weighted mutual information and relative redundancy reduction metrics and ii) the practical similarity between sparsity and redundancy for the systems considered here. We have expanded the figure with additional panels highlighting the three components and expanded our discussion of this result.

20) Is the network fully deterministic, or is neurotransmission stochastic like in the single cell model?

The network simulations also receive inputs from stochastic synapses. This was already described in the Methods subsection 'Perforant path to granule cells' and we have now clarified this in the Results.

21) Line 582: The network lacks many important features, such as feedforward inhibition, interneuron diversity, and mossy-cell effects on inhibition and through their extra-laminar connections. Perhaps most relevant to the conclusion of the papers, the abGC connectivity is not very realistic (should be only weakly coupled to feedback inhibition (cf Temprana and Schinder 2015). The network is also quite small. Because of these limitations, it's not clear what meaning or weight to put in the results shown in Fig 5. These limitations should be acknowledged and discussed.

We agree that our network simulation lacks important features of real dentate gyrus microcircuits, and we look forward to using our new metrics (also available to other researchers in our toolbox) to more exhaustively assess the impact of such features on pattern separation. We have added further acknowledgement of these limitations in the Results and Discussion. We have also added an additional Results section (around the new Figure 7) showing the application of our new metrics to larger models with varied properties.

22) I understand that the goal is primarily to show how to use the new metrics in different settings and that the network simulated is for a proof of concept. But it would help the authors' case to show how interesting conclusion can be drawn from their analysis. To that end, maybe it'd be useful to show how they could also be adapted to spatiotemporal patterns (e.g. like in Yim and Wolfart 2015 but with longer spiketrains)? (see comment #14 and 15). Actually, a direct comparison with Yim and Wolfart 2015 or Madar et al. 2021 would be interesting. They could more directly show in what way their new metrics adds value in comparison to past analyses.

We agree that such a direct comparison would be interesting, but argue it is beyond the scope of our current manuscript which is intended to introduce, justify, and demonstrate our new measures in a number of different circumstances. A valid comparison would require the application of the measures to simulations in models of Madar et al and Yim and Wolfart which we have not implemented in our code.

23) Fig S2E-H: The densities don't match the scatter plots perfectly... What is the first value of similarity shown? (e.g. in Fig S2G top left, there's a high density above 0.3 not seen in FigS2E. Similarly, density goes above 800 for bottom panel, but not in corresponding scatter plot)

We have corrected the axes on this figure, the density plots were correct.

Minor comments:
1) Author summary: "Traditionally, pattern separation has been described alongside the downstream networks that receive the separated representations" : Phrasing is not clear. I gather rom the rest of the paper that it's related to the argument about pattern separation being considered without reference to pattern completion.

We meant to describe the fact that in the original Marr and Albus papers, pattern separation is introduced as a useful precursor to a circuit that does pattern completion. We have clarified this sentence by referencing pattern completion explicitly.

2) Line 47: "increased"? Not clear what the measure is.

This was intended to cover different potential measures and we immediately afterwards give examples of the Hamming and Wasserstein distances. We have rearranged the sentence to be clearer.

3) Line 50: could cite Kreutz papers that introduced raw spike time metrics used in Madar et al. 2019b. Cutts and Eglen 2014 could be cited too, with their STTC metric on raw spike times.

We have added a citations to the 2013 Kreuz et al and 2014 Cutts & Eglen papers here.

4) Line 56 "later studies to use…": grammar?

We have corrected this to 'later studies that used...'

5) Line 123: could add other refs: O'Reilly and McClelland 1994, SeveraAimone2016, Cayco-Gajic and Silver 2019, Madar et al 2019a and 2021).

We have added these citations.

6) Line 213-215 is an exact repetition of line 191-193.

We have removed the second sentence.

7) Line 214 "Here this is idea": typo

We have corrected this.

8) Line 234-5: The phrasing is a bit misleading. The fact that single cells contribute to pattern separation is not in opposition to the role of circuit structure. The single-cell recordings in Madar 2019 were not made in isolation from the network and, in fact, the Madar studies show that pattern separation evaluated at the single cell level is not fully dependent on intrinsic cell properties but strongly dependent on network mechanisms (inhibition, short-term synaptic plasticity).

We have rephrased this to better explain the experimental set-up and findings.

9) Line 291: I find the Hainmueller 2020 review confused on the matter of pattern separation. Mechanisms of pattern separation for population codes are best reviewed in Cayco-Gajic and Silver 2019. The Madar studies show experimental evidence for network mechanism at the single cell level.

We found the Hainmueller 2020 review to contain more useful biophysical details on the mechanisms of pattern separation specifically in the dentate gyrus so have kept this citation but added one to the Cayco-Gajic review.

10) By the way, in the bibliography, 2 different Madar et al 2019 are listed. These should be distinguished as 2019a and b in the main text, for clarity.

We have updated the citations in the main text, as well as for the two Beining et al (2017) papers.

11) Figure 5D: The colour code is confusing because it is the same as in panel C, where blue and grey correspond to differences in input frequency not input phase-modulation strength.

We have changed the colour code for these two panels.

12) Lines 495 and 499: I am not an expert in information theory, but shouldn't the number of words depend on the word length w? (r+1)^w?

For the rate-based codes described here, the information is assumed to be encoded by the rate at a given time (ie bin), so w *would always be 1 in this case. It is of course also possible to extend the coding space to consider information encoded by the modulation of firing rates, where* w>1. *We have clarified this point in the text.*
