# Supplementary material for: Robust and consistent measures of pattern separation based on information theory and demonstrated in the dentate gyrus
Source: PLoS Comput Biol. 2024 Feb 20;20(2):e1010706. doi: 10.1371/journal.pcbi.1010706 (PMC10906873; doi:10.1371/journal.pcbi.1010706)
Supplement: Attachment — Submitted filename: PeerReview_BirdJedlicka_Round2.pdf [file pcbi.1010706.s010.pdf]

The authors have improved their manuscripts in several important ways: 1) their selection of key results in Fig 1 makes it much easier to digest, 2) they added some plots to present how the different components of their new information-theory metrics contribute to the final value, making the interpretation much easier, 3) they show that their redundancy-based metric predicts completion accuracy in a Hopfield network, thus showing that it measures accurately the trade-off between separation and completion and is valuable to find the parameters optimizing that trade-off, 4) they added analysis on a large network of spiking point neurons with grid-cell inputs, showcasing their toolbox for pattern separation measure in yet another context.
I think the study fulfills the stated goal of the authors to introduce their methodology and toolbox to the community. I look forward to trying their toolbox on my data and to reading about their future findings based on those metrics. I only have minor comments and criticism, discussed below.

Main comments (line numbers correspond to the marked-up version, i.e. where changes were identified):

1) The "principle" of information transfer optimization to select binsizes and neural codes is stated here and there but generally conflated with the new metrics as "the information theoretic approach" (e.g., line 360).

a) The authors should highlight, early on, that their approach has two independent components: the new metrics on one hand (which require a choice of binsize and neural code to look at) and the optimization procedure to select the neural code on the other. In the current text, it sometimes reads as if an advantage of the new metrics is to solve the issue of what neural code to investigate, but the metrics presented actually do not intrinsically solve that issue, it's the information transfer optimization that solves it.

b) Like discussed in my first review, I am not convinced that the optimization principle is obviously "the best approach", although I find the idea interesting. Allowing the code and binsize to be different for inputs and outputs is also an interesting and not trivial choice. But such "principle" should be justified biologically, citing the evidence that the brain, and EC and DG in particular, aim to optimize information transfer. Efficient neural coding is a popular theory, but it's by no means definitely proven, the evidence being hard to acquire, especially in the hippocampus. Moreover, the strategy of optimizing for a single code ignores the possibility of multiplexing, which is also a popular idea.

c) Line 361 ("agnostic about the nature…"): Not sure what is meant here. The authors' new metrics still assumes a specific neural code that will be read by downstream networks, no? How is that different from "classical" approaches? Even if you include, in your approach, the principle to optimize the binsize and code to maximize info transfer, it is in itself a non-agnostic choice. For instance, you are still arbitrarily assuming that only one code is relevant, in contrast to the possibility of multiplexed codes. Moreover, the idea that the brain maximizes information transfer is plausible but it is not agnostic. Finally, an infinity of codes exist, and the authors have obviously not included all of them in their optimization procedure, so there is some arbitrary decision in the range of codes explored.

d) The reader is often kept in the dark as to what code and binsize were selected in the end for each analysis. This information should be provided. (I particularly missed that info line 279, 415, 459…)

2) Line 235-6: Unclear phrasing. Please detail why sparsity is considered a proxy for pattern separation. Is it because there is a positive correlation shown in Fig 1C? As pointed out by many papers cited in the present study, sparsity is indeed a mechanism of pattern separation for population codes or temporal codes, but the authors show there are exceptions in Fig 1D and S1 (the Wasserstein distance measure takes a maximum at an intermediate value of the refractory filter, and the decorrelation measure takes a maximum at an intermediate value of the competitive filter where spike trains inhibit each other). Overall, I still think that if one wants to measure pattern separation, they should measure directly pattern similarity and not sparsity, even if the 2 concepts are related.

3) Line 240-2: The justification for gammaM as a metric relevant to pattern separation is clearer in the rebuttal. ("This is because a sparser code that retains information is likely to be using an encoding of higher dimensionality (one of the ideas behind the compressed sensing papers we discuss), and this higher dimensionality causes patterns to separate, as argued in Cayco-Gajic & Silver (2019)."). However, I'm not fully convinced because the argument is based on papers studying population codes and I'm not sure the generalization to temporal or spatiotemporal codes is obvious.
Relatedly, the authors make the claim (line 283), that "The most useful single measure of pattern separation efficacy is gammaM". I do not see why: the redundancy reduction is the only true new pattern separation metric they provide. The authors should make their argument explicit here, or change their claim.

4) Related to point 3, line 522 ("mutual info alone would be a better measure…": Not clear why. The authors need to detail their argument.
I think this stems from the authors' comment in the rebuttal on a hypothetical circuit generating purely random patterns in response to inputs: "*What could the function of such a circuit be, no matter how well separated the outputs are? It would not have a role in the indexing theory of Teyler & DiScenna, which we discuss further below, as no activity patterns could be reliably indexed. Nor could it be used either to train or recall patterns in a classical completion circuit. We argue that such a circuit would not be a 'pattern separator', but a 'pattern destroyer',*"
I respectfully disagree. A black-box generating a purely random index would work well as a pattern separator in collaboration with a separate completion circuit that can match the random index with the full input pattern. This is precisely the index theory (see Legendy 2017 which explore this idea of DG as a random index generator). As discussed in my initial review, as long as the random index generator (DG) is separate (in time and space) from the completion network (CA3) which also receives the non-separated memory pattern to encode (EC inputs, it works in principle (Treves and Rolls 1992). And in that case, the pattern separation is effectively transferring information about the time of the experience to encode.

5) Line 265-6 + equation 3: So, this is equivalent to the strategy of penalizing classical pattern separation metrics with mutual information loss (mentioned above). How does redundancy and classical similarity measures correlate, for the same assumed neural codes? Or, more to the point, what does Redundancy Reduction (not weighted with sparsity or mutual info) bring vs classical metrics? Is it just another metric? Answering those questions are important to convince readers to use the new approach: the new metric must have additional value to balance the fact that using classical methods allows comparisons with past studies.

6) I still think the interpretation of the results could be fleshed out. The reader is often left to ponder on what the result of the analysis means beyond the fact that the metrics are applicable in this or that condition. As pointed in my first review, showing how the new approach can lead to interesting scientific findings will help the main goal, i.e. to showcase the authors' toolbox.

7) Fig 5: I thank the authors for adding that analysis, I think it improves their argument. This figure is not only interesting in showing that gammaR predicts completion performance, but it also operates on a pure population code. That's very different from the temporal codes investigated elsewhere in the paper, isn't it? This could be emphasized. But it also leaves open the question of whether the rest of the results in the paper, where regions of optimal info transfer and pattern separation are identified, also correlate with optimal recall when temporal or spatiotemporal codes are considered. It may not be as obvious as it sounds because recall of spatiotemporal or temporal patterns is trickier.

Minor comments:

Figure S2 G and H: It looks like the density images are rotated and flipped compared to scatter plots in panels S2E-F.

Figure S6E: I have trouble reading that figure, the x-axis and y-axis labels not being symmetric (on the y-axis label, the top 2 rows are labelled as "densities", which does not inform on the variable looked at in panels in the upper right triangle). Also, you could identify clearly in the figure what is for mature GCs and what is for immature GCs.

Line 59: physiological (typo)

Line 71-2: Madar et al 2019 (PLoS Comp Bio) also has a supplementary discussion on the relationship between sparsity and similarity metrics that builds on and adds to de la Rocha 2007 and Cutts and Eglen 2014.

Line 79-80: The role of noise/information destruction in pattern separation is investigated in the Madar 2019 Scientific Reports article, not the PLOS Comp Biol.

Line 80-89: I understand the authors want to introduce the storied debate about the computational role of immature vs mature GCs, but maybe it’s worth making it more to the point here, because, as is, it is an incomplete review of the literature. For instance, Nakashiba et al. 2012, although influential, does not really pertain to network computation (despite their inaccurate use of the pattern completion/separation vocabulary) but to generalization/discrimination behavior. Moreover, that particular study’s conclusions have been undermined for lacking an essential control experiment (see Piatti and Leutgeb’s 2013 review) and the field has moved beyond (or should have). Referencing recent reviews and papers (e.g. Tuncdemir and Hen 2019, Gozel and Gerstner 2021) may help making the authors point and would be helpful to readers for reference.

Line 197-200: Please provide an interpretation of those exceptions. Why and what does that say?

Line 208: in Fig 1E (and all subsequent analyses), is the optimization procedure (maximizing information transfer over all bins and codes) done separately for each x-axis condition tested? In other words, are the code and binsize potentially different for different

Line 246-7 “there can be value in considering the components separately”): too vague. The authors should make that value explicit, for instance by detailing the interpretation for an example analysis.

Line 277: correlation being phase locking here. This was referred to as "input similarity" elsewhere in the text and figures. Personally, I don’t think using the term “similarity” instead of correlation solves the potential confusion, because a specific kind of similarity measure is implied. I think it’d be best to be precise and say “phase-locking strength” throughout the study, given that many other similarity definitions are employed elsewhere in the study.

Line 307: Note that more work is needed to accurately assess those parameters at EC-DG synapses. Madar et al.’s model was a proof of concept, not really a rigorous attempt at estimating facilitation and depression parameters of the Tsodyks-Markram equations (only 1 cell was fitted). Hippocampome.org also has some estimates for these parameters, worth looking into.

Line 333: A conclusion to interpret the results of Fig 3c is lacking to this paragraph. However, I'm glad the authors plotted those separate components. I now better see the value of combining a pattern separation metric (e.g. RR) with mutual info: it allows to quantify and visualize immediately at which parameters the trade-off is optimized, which is not necessarily obvious when looking at the 2 measures are analyzed separately. The authors should emphasize that point.

Line 377 ("to be the be the"): typo

Line 413: Not clear what to make of the monotonic increase in gammaR in Fig 6c.

Line 421-3: Dieni & Overstreet-Wadiche papers (2013, 2016) are also relevant here, showing that despite increased excitability, immature GCs receive less EC inputs and thus fire sparsely.

Line 441-3: I thought this was a helpful conclusion sentence, that helps the reader understand what the author's goal was.

Line 444-9: I would put this paragraph in the preceding one, before the conclusion sentence. I think Fig 6f definitely helps see how 6e makes sense.

Line 459: Meaning that a pure ensemble code is considered, in contrast to Fig 1? or a spatiotemporal code? In general, clarifying throughout the manuscript which code is considered for a given analysis would be really helpful for the reader (see main comments).

Line 517 ("information rates could be used…"): unclear

Line 577 (use of ratio rather than difference between input and output similarities): Great to highlight that comparisons with other studies may not possible, but I think this is not specific to Madar papers. Most if not all papers on pattern separation I know use a difference rather than ratio. Please cite papers that chose the ratio strategy if this is not an innovation on your part.

Line 817: typo on "neurons"

Line 831: typo on "heterogeneity"
