# Supplementary material for: Robust and consistent measures of pattern separation based on information theory and demonstrated in the dentate gyrus
Source: PLoS Comput Biol. 2024 Feb 20;20(2):e1010706. doi: 10.1371/journal.pcbi.1010706 (PMC10906873; doi:10.1371/journal.pcbi.1010706)
Supplement: Attachment — Submitted filename: Response_Rnd_2.pdf [file pcbi.1010706.s011.pdf]

Reviewer 1:

I think this paper is greatly improved from its original submission, which I enjoyed on my first reading as well. Thank you to the authors for addressing most of my concerns.

One minor technical point / question. In the new Figure 7 on the large scale simulation, the authors vary adult-born fraction from 0 to 1. In biological systems, the percentage of immature neurons is likely between 1% and 10% (depending on animal age and species); so the relevant range to examine is in between the first and second points on that graph. Since these experiments are at near mouse scales, a 1% rate is still meaningful. So it'd be interesting to run experiments where those low fractions of new neurons are examined. While this is a simple model, I'd expect that it is possible to see a non-linear "bump" of sorts with a very low rate of new neurons coming in.

Alternatively, the effect of neurogenesis may be different in different areas of the excitation - inhibition balance. For instance, does the location of the peak in Figure 7C or Figure 7D change if the proportion of new neurons changes?

We would like to thank the reviewer for their feedback. We agree that there is more to study with respect to the influence of adult-born cells on pattern separation and hope to continue this with further studies. We agree that the introduction of a very small amount of heterogeneity might have lead to peak in pattern separation performance for very few adult-born cells. However, when we ran our simulations again, with proportions of adult-born cells between 0% and 10% of the total, we did not observe this in our model and the results confirmed the existing trend of always-decreasing performance. We have added an additional datapoint corresponding to 5% adult-born cells to Figure 7.

We agree that it is likely that changing the proportion of adult-born cells would alter the effects of inhibition and would expect that more adult-born cells would imply peak pattern separation performance at higher levels of inhibition. We have seen signs of this effect in exploratory simulations, but have not included full results in the manuscript as we do not think they add enough insight without more careful control of the connectivity to the adult-born cells. However, to take into account this relevant point of the reviewer, we have added this information on p21 of the revised manuscript.

Reviewer 2:
The authors have improved their manuscripts in several important ways: 1) their selection of key results in Fig 1 makes it much easier to digest, 2) they added some plots to present how the different components oftheir new information-theory metrics contribute to the final value, making the interpretation much easier, 3) they show that their redundancy-based metric predicts completion accuracy in a Hopfield network, thus showing that it measures accurately

the trade-off between separation and completion and is valuable to find the parameters optimizing that trade-off, 4) they added analysis on a large network of spiking point neurons with grid-cell inputs, showcasing their toolbox for pattern separation measure in yet another context.

I think the study fulfills the stated goal of the authors to introduce their methodology and toolbox to the community. I look forward to trying their toolbox on my data and to reading about their future findings based on those metrics. I only have minor comments and criticism, discussed below.
We would like to thank the reviewer for their many helpful comments that have helped us improve the manuscript. We are glad that the reviewer is interested in our work and in using our new tools. We address their comments below.

Main comments (line numbers correspond to the marked-up version, i.e. where changes were identified):

1) The "principle" of information transfer optimization to select binsizes and neural codes is stated here and there but generally conflated with the new metrics as "the information theoretic approach" (e.g., line 360).

a) The authors should highlight, early on, that their approach has two independent components: the new metrics on one hand (which require a choice of binsize and neural code to look at) and the optimization procedure to select the neural code on the other. In the current text, it sometimes reads as if an advantage of the new metrics is to solve the issue of what neural code to investigate, but the metrics presented actually do not intrinsically solve that issue, it's the information transfer optimization that solves it.
We agree that this is unclear. Our approach in optimising over codes and binsizes is in line with what we believe to be best practice in applying information theory to neuronal data (see eg Timme & Lapish, 2018), but it is not universally applied. It is helpful to emphasise our approach here and we have added the following sentence when we introduce our new toolbox: 'This toolbox combines both the calculation of the new metrics themselves, which depend on choices of neural code and numerical parameters such as discretisation binsize, and the optimisation algorithms we use to identify the codes and parameters that allow the maximum amount of information to be decoded'.

b) Like discussed in my first review, I am not convinced that the optimization principle is obviously "the best approach", although I find the idea interesting. Allowing the code and binsize to be different for inputs and outputs is also an interesting and not trivial choice. But such "principle" should be justified biologically, citing the evidence that the brain, and EC and DG in particular, aim to optimize information transfer. Efficient neural coding is a popular theory, but it's by no means definitely proven, the evidence being hard to acquire,

especially in the hippocampus. Moreover, the strategy of optimizing for a single code ignores the possibility of multiplexing, which is also a popular idea.
We agree that multiplexing is an interesting idea that we do not explore in enough detail here. We have added a suggestion of how our measures might be extended to this case in the Discussion. Our choice of optimising the code to maximise the potential information transmission (including the choice of allowing separate timescales for the inputs and outputs) is to allow us to consider the maximum amount of information that could be reconstructed from a neural signal (over the set of codes and parameters we consider). This is not quite the same as assuming that the EC-DG circuit aims to optimize information transfer as in Severa et al (2017), and we now make this clear.

c) Line 361 ("agnostic about the nature…"): Not sure what is meant here. The authors' new metrics still assumes a specific neural code that will be read by downstream networks, no? How is that different from "classical" approaches? Even if you include, in your approach, the principle to optimize the binsize and code to maximize info transfer, it is in itself a non-agnostic choice. For instance, you are still arbitrarily assuming that only one code is relevant, in contrast to the possibility of multiplexed codes. Moreover, the idea that the brain maximizes information transfer is plausible but it is not agnostic. Finally, an infinity of codes exist, and the authors have obviously not included all of them in their optimization procedure, so there is some arbitrary decision in the range of codes explored.
The agnosticism relates to the structure of the downstream pattern completion circuit. We assume that it is better for that circuit to receive an input that would allow it to recover more information about the inputs to the pattern separation circuit (again, over the set of codes we consider), but we do not worry about how exactly it decodes that information. We acknowledge more clearly that although we consider multiple codes, it is not possible to consider all possible ways that information could be encoded (p12).

d) The reader is often kept in the dark as to what code and binsize were selected in the end for each analysis. This information should be provided. (I particularly missed that info line 279, 415, 459…)
The optimal binsizes (and sometimes codes) are typically different for each datapoint, and typically differ between the input and output ensembles. It is not trivial to summarise these for the figures plotted. We have clarified this before (the old) line 279.

2) Line 235-6: Unclear phrasing. Please detail why sparsity is considered a proxy for pattern separation. Is it because there is a positive correlation shown in Fig 1C? As pointed out by many papers cited in the present study, sparsity is indeed a mechanism of pattern separation for population codes or temporal codes, but the authors show there are exceptions in Fig 1D and S1 (the Wasserstein distance measure takes a maximum at an intermediate value of the refractory filter, and the decorrelation measure takes a maximum at an intermediate value of the competitive filter where spike trains inhibit each other). Overall, I still think that if one wants to measure pattern separation, they should measure directly pattern similarity and not sparsity, even if the 2 concepts are related.

We agree that sparsity itself is not pattern separation (although it tends to be highly correlated with more specific measures). Our argument here is directly related to that below: combining sparsity with mutual information implies a higher dimensional code in which patterns can be more reliably separated. We also acknowledge here the possibility of combing mutual information with, for example, orthogonalisation. We have made the fact that sparsity should always be considered alongside mutual information in the context of pattern separation clearer (below Eq 1, p11).

3) Line 240-2: The justification for gammaM as a metric relevant to pattern separation is clearer in the rebuttal. ("This is because a sparser code that retains information is likely to be using an encoding of higher dimensionality (one of the ideas behind the compressed sensing papers we discuss), and this higher dimensionality causes patterns to separate, as argued in Cayco-Gajic & Silver (2019)."). However, I'm not fully convinced because the argument is based on papers studying population codes and I'm not sure the generalization to temporal or spatiotemporal codes is obvious. Relatedly, the authors make the claim (line 283), that "The most useful single measure of pattern separation efficacy is gammaM". I do not see why: the redundancy reduction is the only true new pattern separation metric they provide. The authors should make their argument explicit here, or change their claim.
We do think that this argument applies to codes of different types. Although the Cayco-Gajic & Silver paper studied population codes, the Ganguli & Sompolinksy paper is more abstract as to how a signal is encoded and provides a solid argument for this generalisation to other code types. Regarding the second point, the authors have found in discussion with colleagues that upsilonM is a useful way to introduce the concept of including information throughput in pattern separation calculations, and that upsilonR can appear more esoteric. We have changed the wording here to 'Upsilon_M is a simple to understand and easy to apply measure of pattern separation...'.

4) Related to point 3, line 522 ("mutual info alone would be a better measure…": Not clear why. The authors need to detail their argument. I think this stems from the authors' comment in the rebuttal on a hypothetical circuit generating purely random patterns in response to inputs: "What could the function of such a circuit be, no matter how well separated the outputs are? It would not have a role in the indexing theory of Teyler & DiScenna, which we discuss further below, as no activity patterns could be reliably indexed. Nor could it be used either to train or recall patterns in a classical completion circuit. We argue that such a circuit would not be a 'pattern separator', but a 'pattern destroyer'," I respectfully disagree. A black-box generating a purely random index would work well as a pattern separator in collaboration with a separate completion circuit that can match the random index with the full input pattern. This is precisely the index theory (see Legendy 2017 which explore this idea of DG as a random index generator). As discussed in my initial review, as long as the random index generator (DG) is separate (in time and space) from the completion network (CA3) which also receives the non-separated memory pattern to encode (EC inputs, it works in principle (Treves and Rolls 1992). And in that case, the pattern separation is effectively transferring information about the time of the experience to encode.

This comment is indeed a direct response to the indexing theory. It is possible that we have been using a different interpretation of the idea of 'randomness' to the reviewer. From our perspective, a completely random index would mean that there would be no information between the input and the output, and a downstream pattern completion circuit, no matter how well separated in time or space, would not be able to usefully reconstruct anything about the input or its time. A circuit that produces outputs that appear very different to the inputs, but contain mutual information about them, would not be 'random' in our conception. It is possible for mutual information to be computed between different domains, such as neural activity and behaviour, and the randomness would depend on the measured mutual information, not the difference between the domains. We stand by our original comment about the role of a purely random circuits (where, crucially the output is independent of the input and contains no information about it). In this case, it would not be possible to match the purely random input to any pattern of activity. To quote from the Legendy 2017 paper:

'recognition based on samples can only be reliable when the samples are representative. In the present case, this means that (ideally) the samples must contain enough elements from every cell assembly of the ensemble to make them recognizable, which in turn means that the samples, wherever they are taken, must contain enough information (entropy) to permit recognition to an acceptable level of certainty'.

The situation where a purely random circuit might be useful would be when the dentate gyrus generates randomness to help construct distributed cell assemblies in CA3, but subsequently has no role in recall, with some other circuit generating reliable yet sparse inputs when recall is required (maybe direct connections to CA3). In this case, it would be futile to measure pattern separation in the dentate gyrus at all. We have expanded our Discussion to include this idea (p23-24).

5) Line 265-6 + equation 3: So, this is equivalent to the strategy of penalizing classical pattern separation metrics with mutual information loss (mentioned above). How does redundancy and classical similarity measures correlate, for the same assumed neural codes? Or, more to the point, what does Redundancy Reduction (not weighted with sparsity or mutual info) bring vs classical metrics? Is it just another metric? Answering those questions are important to convince readers to use the new approach: the new metric must have additional value to balance the fact that using classical methods allows comparisons with past studies.
Redundancy reduction alone is indeed a measure of pattern separation, and we would consider it to be particularly useful as it retains many of the features of the relative measure upsilonR in being more robust to specific features of the input due to the ability to optimise over codes and parameters. We would not, however, aim to promote it as a pattern separation metric in its own right as it shares the key issue of neglecting information throughput with the classical measures. As the reviewer points out, losing comparability with past results is unfortunate and would require the substantial improvements

we feel upsilonM and upsilonR bring to justify. We have added an explanation of this when we introduce the redundancy reduction (p12).

6) I still think the interpretation of the results could be fleshed out. The reader is often left to ponder on what the result of the analysis means beyond the fact that the metrics are applicable in this or that condition. As pointed in my first review, showing how the new approach can lead to interesting scientific findings will help the main goal, i.e. to showcase the authors' toolbox.
We have expanded our discussion of the implications of short-term synaptic plasticity (around Figure 3, p13), and the balance between Kir2 channel expression and synaptic potentiation in the context of epilepsy (around Figure 6, p19). We think that both of these results are interesting scientific findings that are not accessible to standard pattern separation metrics (see Fig S4, and upper panels 7B-E). We hope that such results will highlight the utility of our new metrics.

7) Fig 5: I thank the authors for adding that analysis, I think it improves their argument. This figure is not only interesting in showing that gammaR predicts completion performance, but it also operates on a pure population code. That's very different from the temporal codes investigated elsewhere in the paper, isn't it? This could be emphasized. But it also leaves open the question of whether the rest of the results in the paper, where regions of optimal info transfer and pattern separation are identified, also correlate with optimal recall when temporal or spatiotemporal codes are considered. It may not be as obvious as it sounds because recall of spatiotemporal or temporal patterns is trickier.
We agree that the demonstration of upsilonR predicting pattern completion performance is a useful addition and thank the reviewer for their original suggestion. However, we also consider population codes to some extent in the following figures 6 and 7. We have now highlighted this (p18). We agree that our results here are not proof that upsilonR computed on any code is correlated with improved pattern recall by some downstream circuit, and now acknowledge this more clearly in this Results section (p18).

Minor comments:
Figure S2 G and H: It looks like the density images are rotated and flipped compared to scatter plots in panels S2E-F.
There was an error in the code plotting the heatmaps (the x and y axes were flipped) and this has been corrected. We would like to thank the reviewer for noticing this.

Figure S6E: I have trouble reading that figure, the x-axis and y-axis labels not being symmetric (on the yaxis label, the top 2 rows are labelled as "densities", which does not inform on the variable looked at in panels in the upper right triangle). Also, you could identify clearly in the figure what is for mature GCs and what is for immature GCs.

We have added titles for each marginal and have labelled the mature and immature sections.

Line 59: physiological (typo)
We have corrected this.

Line 71-2: Madar et al 2019 (PLoS Comp Bio) also has a supplementary discussion on the relationship between sparsity and similarity metrics that builds on and adds to de la Rocha 2007 and Cutts and Eglen 2014.
We have acknowledged this.

Line 79-80: The role of noise/information destruction in pattern separation is investigated in the Madar 2019 Scientific Reports article, not the PLOS Comp Biol.
We have corrected this.

Line 80-89: I understand the authors want to introduce the storied debate about the computational role of immature vs mature GCs, but maybe it's worth making it more to the point here, because, as is, it is an incomplete review of the literature. For instance, Nakashiba et al. 2012, although influential, does not really pertain to network computation (despite their inaccurate use of the pattern completion/separation vocabulary) but to generalization/discrimination behavior. Moreover, that particular study's conclusions have been undermined for lacking an essential control experiment (see Piatti and Leutgeb's 2013 review) and the field has moved beyond (or should have). Referencing recent reviews and papers (e.g. Tuncdemir and Hen 2019, Gozel and Gerstner 2021) may help making the authors point and would be helpful to readers for reference.
We would like to thank the reviewer for these suggestions. We have clarified that the findings of Nakashiba et al concern behavioural tasks and summarised and cited the other three studies. In the revised manuscript, we have also cited a new review/discussion paper on the general role of the dentate gyrus (Borzello et al. Nat Neuroci 2023).

Line 197-200: Please provide an interpretation of those exceptions. Why and what does that say?
We have added a brief interpretation of these exceptions: 'As the Wasserstein distance is rate-invariant (normalised to the total number of spikes in each train), it appears that removing spikes that lie close to other spikes can lead to lower measured distances \citep{Sihn2019}. For the decorrelation measure, the competitive filter removes spikes close to others in all parts of the ensemble and so can lead to very sparse vectors that are actually more correlated in the chosen time windows.'

Line 208: in Fig 1E (and all subsequent analyses), is the optimization procedure (maximizing information transfer over all bins and codes) done separately for each x-axis condition tested? In other words, are the code and binsize potentially different for different.

Yes, the codes and binsizes are optimised for each condition. We now clarify this.

Line 246-7 “there can be value in considering the components separately”): too vague. The authors should make that value explicit, for instance by detailing the interpretation for an example analysis.
We have added ‘to see how far pattern separation performance is driven by maintenance of information throughput and how far by reduction in input similarity (see Results below)’ here and already discuss an example analysis for Figure 3C.

Line 277: correlation being phase locking here. This was referred to as "input similarity" elsewhere in the text and figures. Personally, I don’t think using the term “similarity” instead of correlation solves the potential confusion, because a specific kind of similarity measure is implied. I think it’d be best to be precise and say “phase-locking strength” throughout the study, given that many other similarity definitions are employed elsewhere in the study.
We have clarified that the similarity shown in Figure 3 here is phase-locking strength, but that the effect is more general and holds across the other types of similarity (auto- and cross-correlations) that we use elsewhere.

Line 307: Note that more work is needed to accurately assess those parameters at EC-DG synapses. Madar et al.’s model was a proof of concept, not really a rigorous attempt at estimating facilitation and depression parameters of the Tsodyks-Markram equations (only 1 cell was fitted). Hippocampome.org also has some estimates for these parameters, worth looking into.
We would like to thank the reviewer for this suggestion and have added the consensus Hippocampome.org values here. The average parameter estimates are currently very similar to those originally cited.

Line 333: A conclusion to interpret the results of Fig 3c is lacking to this paragraph. However, I’m glad the authors plotted those separate components. I now better see the value of combining a pattern separation metric (e.g. RR) with mutual info: it allows to quantify and visualize immediately at which parameters the trade-off is optimized, which is not necessarily obvious when looking at the 2 measures are analyzed separately. The authors should emphasize that point.
We would like to thank the reviewer for this suggestion and have added this conclusion.

Line 377 (“to be the be the”): typo
We have corrected this.

Line 413: Not clear what to make of the monotonic increase in gammaR in Fig 6c.
We typically find that upsilonR is sensitive to the degree of input correlation and now explain this in the results.

Line 421-3: Dieni & Overstreet-Wadiche papers (2013, 2016) are also relevant here, showing that despite increased excitability, immature GCs receive less EC inputs and thus fire sparsely.
We also found these references useful and now cite them here.

Line 441-3: I thought this was a helpful conclusion sentence, that helps the reader understand what the author's goal was.
We have re-ordered this paragraph as suggested below to demonstrate which aspects of pattern separation are maintained by the changes considered, and how their interaction might lead to similar functionality despite epileptic-type changes..

Line 444-9: I would put this paragraph in the preceding one, before the conclusion sentence. I think Fig 6f definitely helps see how 6e makes sense.
See response above.

Line 459: Meaning that a pure ensemble code is considered, in contrast to Fig 1? or a spatiotemporal code? In general, clarifying throughout the manuscript which code is considered for a given analysis would be really helpful for the reader (see main comments).
We have clarified that a population/ensemble rate code is considered here.

Line 517 ("information rates could be used…"): unclear
We have clarified this by replacing this sentence with 'When pattern separation performance is believed to change over time, the rate of information transmission at a given time (in bits/s) could be calculated instead of the total mutual information (in bits) in our new measures.'

Line 577 (use of ratio rather than difference between input and output similarities): Great to highlight that comparisons with other studies may not possible, but I think this is not specific to Madar papers. Most if not all papers on pattern separation I know use a difference rather than ratio. Please cite papers that chose the ratio strategy if this is not an innovation on your part.
We have added a brief summary of approaches taken by other studies here.

Line 817: typo on "neurons"
We have corrected this.

Line 831: typo on "heterogeneity"

We have corrected this.
