# Supplementary material for: Robust and consistent measures of pattern separation based on information theory and demonstrated in the dentate gyrus
Source: PLoS Comput Biol. 2024 Feb 20;20(2):e1010706. doi: 10.1371/journal.pcbi.1010706 (PMC10906873; doi:10.1371/journal.pcbi.1010706)
Supplement: Attachment — Submitted filename: Response_Rnd_3.pdf [file pcbi.1010706.s012.pdf]

We would like to thank the reviewer for their careful reading of our manuscript and many helpful comments, which we address individually below.

Reviewer 2:

1) Upsilon_M as a measure of Pattern Separation (Line 256 and 291 in the marked-up manuscript): Despite the authors' response to my comments #2 and #3, I still think it is erroneous to call Upsilon_M a measure of pattern separation, as it does not directly compare measures of pattern similarity. Even if it necessarily correlates with pattern separation (which is not demonstrated in the paper), that's not what it measures. It measures efficient coding (sparse and informative). The argument that sparse information transmission is equivalent to pattern separation in a high dimensional space is a very interesting idea, but this argument is not explicitly laid out in the manuscript (only partially in the rebuttal). A citation of Ganguli and Sompolinski 2012 (which does not directly talk about pattern separation) is not enough: if the authors want to claim (and convince) that their metric is the better way to think about pattern separation, they need to make a rigorous demonstration that there is an equivalence or a correlation. And, at the very least, instead of calling it a "measure of pattern separation", I think it would be safer to call Upsilon_M a "proxy" for pattern separation. The field of pattern separation is already ridden with confusing terminology applied differently in different context, best not to add to the confusion.

We agree that Upsilon_M is a less direct measure of pattern separation than many classical measures. We have made the following changes to the text to highlight this:

- *'We note again here that sparsity on its own is not a measure of pattern separation, but can be combined with mutual information I(X,Y) to produce one.' →'We note again here that sparsity on its own is not a measure of pattern separation, but can be combined with mutual information I(X,Y) to produce a useful proxy.'*
- *'Upsilon_M is a simple to understand and easy to apply measure of pattern separation' → 'Upsilon_M is a simple to understand and easy to apply proxy for pattern separation'*

2) Reporting the codes and binsizes selected by the optimization procedure (response to comment 1d in previous review + see line 284 of the marked-up manuscript):
I understand that this information is not easy to summarize. Still important to report here, to provide the reader with a full understanding of the data presented in the manuscript. The full info (not summarized) could be made available as a supplementary table. For a more palatable summary, one could imagine a plot of the distribution of binsizes over all analyses (one plot for inputs, one for outputs), to see if they vary widely or if we always end up in the same ballpark. And for some of the most important analyses (e.g. Fig 3 and 6), the authors could at least report the neural codes and binsizes that maximized the used metric. These are just ideas, there might be better ones, the goal being to make the optimization procedure less of a black-box to the reader. There is some interesting info there.

We are still in the process of compiling the full raw codes and sizes into .csv tables and are uploading them to the Zenodo data repository (doi 10.5281/zenodo.10233025). Unfortunately, the processing is now much slower due to saving these extra values and the full raw data output has not finished yet. We hope not to delay publication of this manuscript further and will continue uploading the remaining data as it finishes. In uncompressed form, the data tables take up several gigabytes of storage space and we do not think it would be appropriate to include this as a printed table. Summarising bin sizes can be difficult, particularly in the later analyses as the optimal codes can differ and, for example, a rate-based code with 100ms bins is not obviously comparable to a spike-timing code with 100ms bins.

Minor comment:
Line 602-4 (in marked-up manuscript), the authors added more comparison on methods used to measure pattern separation in past studies (use of a ratio vs a difference between input and output similarities). I think some terms used are too vague (it is hard to relate to the problem of ratio vs difference of pattern separation) and sometimes inaccurate: 1) not sure what is meant by "overlap" (in reference to the Stark review). Overlap is a

measure of similarity, not of pattern separation, isn't it?, 2) "integrated over a range", in reference to the Guzman study: not clear a range of what? How does that relate to the ratio vs difference approaches? 3) As far as I understand, the Yim and Wolfart 2014 study does not use ratios but plots input vs output similarities, like in the Madar studies. Most studies or analyses actually do not make a decision in this ratio vs difference debate, they just plot the similarities, allowing a direct visual comparison.

1) not sure what is meant by "overlap" (in reference to the Stark review). Overlap is a measure of similarity, not of pattern separation, isn't it?,

The Yassa & Stark review uses the concept of input pattern overlap increasing (pattern completion) or decreasing (pattern separation). We have added a 'decreased' before 'overlap' to clarify this.

2) "integrated over a range", in reference to the Guzman study: not clear a range of what? How does that relate to the ratio vs difference approaches?

This Guzman et al study takes an integral of output correlation minus input correlation over a range of input correlations to produce a single measure of pattern separation that is independent of input correlation. We have clarified this by adding 'over a range of different inputs (Guzman et al, 2021). The latter approach removes the influence of input similarity on the measure, but requires more simulation time.'

3) As far as I understand, the Yim and Wolfart 2014 study does not use ratios but plots input vs output similarities, like in the Madar studies. Most studies or analyses actually do not make a decision in this ratio vs difference debate, they just plot the similarities, allowing a direct visual comparison.

We agree that the Yim and Wolfart paper plots the input against output similarities, but also fits power laws to this data as a way to quantify the changes in pattern separation performance due to mossy fibre spouting and changing leaks (Figure 1e shows the fit, 6c shows the single measure in that paper). We have clarified this in the text, replacing this sentence with: 'Studies such as (Yim, et al 2015) plot both input and output similarities, whilst fitting ratios as here in order to

quantify changes in pattern separation performance. Such an approach retains the benefit of presenting both input and output similarities separately for direct visual comparison, a common feature in pattern separation studies, but also provides a single metric to directly measure changes in performance.'
