# Supplementary material for: Robust and consistent measures of pattern separation based on information theory and demonstrated in the dentate gyrus
Source: PLoS Comput Biol. 2024 Feb 20;20(2):e1010706. doi: 10.1371/journal.pcbi.1010706 (PMC10906873; doi:10.1371/journal.pcbi.1010706)
Supplement: Attachment — Submitted filename: PeerReview_BirdJedlicka2022_PlosCompBiol.docx [file pcbi.1010706.s008.docx]

**Summary:**

This manuscript makes the case for including considerations about information transmission when studying pattern separation in the context of episodic memory and dentate gyrus processing. A systematic analysis of different types of similarity metrics confirms and extends past results that sparsity and information-destroying unreliability are sufficient to perform pattern separation. The authors then introduce 3 new metrics (and ways to estimate them with sparse data), based on common information-theory measures (mutual information, transfer information or redundancy). These metrics are designed with the goal of detecting pattern separation that preserves information. The use of these new metrics is displayed in different contexts: a biophysically realistic single granule cell model receiving cortical inputs, and a small model of the dentate gyrus network with normal or pathological parameters. The analysis of the single GC simulations with the new metrics 1) confirms that different facilitation and depression parameters yields different pattern separation levels, 2) identifies short-term dynamics that maximize information transmission, and 3) shows that conductance heterogeneity does not impact pattern separation, confirming the degeneracy of these parameters. The analysis of the network simulations confirms that different cell-types can impact separation and information transmission differently, and suggests that the pathological increase of input strength and intrinsic excitability observed in epilepsy can both lead to information loss and decreased separation, which is new and interesting but may be confounded by the way the new metrics are designed.

I think the use of information-theory approaches to better understand dentate computations and pattern separation in general is a valuable endeavor. The methodology is rigorous, and the presentation of the results is neat, relatively clear and put in the context of the relevant literature, but the interpretation of the results needs to be fleshed out for the reader to learn how to interpret the new metrics and to better understand the significance of the proposed methods. My main concern is with the design of the new metrics. In particular: 1) I am not convinced that the sparsity-weighted mutual information is measuring pattern separation, 2) I think combining information transmission with similarity measures creates interactions that are difficult to interpret and that both aspects of a networks’ computation are best analyzed separately.

**Comments:**

1) Line 33-77: The 2nd paragraph of the introduction is both 1) a presentation of past strategies to measure pattern separation and 2) a motivation for the study by showing problems in the current way pattern separation is measured. I think conflating the 2 points is confusing. To make things clearer, I suggest separating the review of past similarity metrics (they all have their pros and cons and assumptions, including different dependencies to sparsity and firing rates), from the motivation for the need to better control for information loss when studying pattern separation.

1a) One potential problem with similarity metrics is their dependency over firing rates or network sparsity. This is touched upon diffusely in the manuscript (line 53-57, line 98-100, line 123-4, line 197, line 449) and some of the confusion might be reduced if it was addressed separately from other issues or motivations. This point seems particularly relevant to results in Figure 1 (paragraph starting line 118) and to explaining the “sparsity-weight” used in the design of the new information-theory metrics. I also think it should be noted that most past studies cited on the role of sparsity in pattern separation (e.g. line 123) have considered a binary population code, and thus network sparsity rather than spiking sparsity as is the case in this study. Cutts and Eglen 2014 (not cited) and Madar et al 2019b (cited), seem most relevant to the present study, as they both have interesting discussions on the effect of firing rates on similarity metrics for spiketrains.

1b) Line 58: the issue of bin size choice noted for classical similarity metrics remains for the information-theory based metric presented here: these new metrics are not parameter-free and choices must be made concerning the type of neural code investigated and the word and/or letter length.

1c) Line 51, authors point to inconsistent results between studies, but most of these apparent inconsistent claims are first due to differences in what patterns of neuronal activity were considered, so they are not really inconsistencies. Many studies indeed claim to measure “pattern separation” but actually refer to a broad class of computations. This makes the literature confusing, because findings are really about different neural codes and computations. In this paragraph, authors have grouped and compared studies that considered either ensemble of binary neurons or spike trains in single neurons, or different types of spatiotemporal patterns. This unaddressed diversity makes the authors argument somewhat difficult to follow and potentially misleading, as the pros and cons of each metric won’t be the same or mean the same thing for different types of activity patterns.

1d) Line 65, it is said that Vineyard 2016 and Madar 2019 explicitly note that the fact that information-destructing noise can lead to high pattern separation levels is a limitation of standard metrics. That is not my reading of the literature: the metrics are not intrinsically at fault and those papers do not make that claim. Pattern separation is not defined in terms of information, so it may (or may not) be performed by purely random processes, and the papers cited note that trade-off between pattern separation and reliability of information transmission. Measuring pattern separation and reliability are two different questions: one is quantifying the degree of separation and the other is about the mechanism underlying the separation (random information-destructive process or not). I agree that both are important to test the role of pattern separation in episodic memory, but the concepts are separate.

2) The authors’ premise and justification for their new metrics is that information destruction is undesirable for pattern completion and episodic memory to work properly.

2a) I do not think that this premise is obviously true. It depends on when pattern separation is performed. Pattern separation is theorized to happen during encoding and pattern completion during recall. When those two computations are separated in time, using an unreliable process to pattern separate during encoding does not then necessarily preclude recall in CA3 at a later time point: DG might not perform pattern separation during recall, or might be decoupled from CA3 during that phase, leaving only the EC input to CA3 to trigger pattern completion (cf Treves and Rolls 1992, O'Reilly and McClelland 1994).

2b) Moreover, noise is not necessarily a bad thing when it comes to pattern separation, and can be a useful feature rather than a bug, for instance promoting robustness or efficiency in population codes (see Wolpert 2008, Hunsberger and Eliasmith 2014 or Washke and Garrett 2021) and even information transfer in some cases (e.g. Goldberg 2004, a paper that the authors cite). As pointed out in Madar et al. 2019a, in the case of episodic memory the unreliability can be meaningful and carry information about the time of the episode being stored. The pattern separation process could even potentially fully destroy all the information content from the cortex and CA3 would just store a randomly-generated index, which corresponds to the original index theory of episodic memory (Teyler and Rudy 2007, Legendy 2017 https://pubmed.ncbi.nlm.nih.gov/28593904/).

2c) Line 34-36 “When considered as part of a system preceding pattern completion, good pattern separation can be simply taken as any procedure that reduces noise and enhances the storage capacity of the downstream network”: As hinted above, the assumption that good pattern separation should “reduce noise” is debatable (especially given the wide array of definitions that the term “noise” can take. However, the fact that the form of pattern separation we are most interested in the context of DG and episodic memory are the ones that help increase storage capacity in CA3 is true and at the center of the authors argument and motivation for designing new pattern separation metrics. So, they should show that their methodology is able to distinguish between “good” and “bad” pattern separation, by showing that high pattern separation levels as assessed by their metric necessarily lead to improved storage capacity without catastrophic interferences.

2d) Similarly, it would strengthen their argument if they could show that information-destructive pattern separation is *necessarily* bad for subsequent pattern completion. I guess this brings us back to the preceding point, as it’s not clear that a separating process that completely destroys the information contained in the inputs would impair storage if the role of the dentate is to generate a random index that informs on the time of the episode (Legendy 2017). DG is not the only source of input to CA3 and is often hypothesized to not be particularly important to trigger pattern completion during the recall phase (Treves and Rolls 1992, Hainmueller and Bartos 2020).

3) All that said, I think using information-theoretic approaches to quantify pattern separation makes a lot of sense. Shannon’s entropy is a natural concept to apply to assess the variability in a group of activity patterns, and whether this variability is reduced in the output.

Line 189: Measuring redundancy in particular does appear as a useful and genuine similarity metric. Moreover, figuring out whether the redundancy in inputs has been removed in the output is an important hypothesis that has been challenging to test. The combination of mutual information measure with redundancy could help. Can such measure identify which parts of the inputs are redundant and check whether the output ensemble retained the non-redundant spikes?

4) Input correlations: could you note in figure 1 that these correspond to the phase-locking strength (as opposed to Pearson’s correlation, or input similarity measured through each plotted metric)? Showing the pattern separation levels for different input correlations is important and interesting, however I see multiple issues or sources of confusion here.

4a) phase-modulation strength is yet another “non-classical” similarity metric used in this paper, which could have been used to measure the input and output similarities.

4b) One generally wants to know how pattern separation levels differ for different input similarity levels measured the same way that output similarity was measured. It would be useful to show that directly, or to show how phase-locking strength relates to the other metrics used.

4c) relatedly, since the plotted pattern separation levels are averages over all pairs of spiketrains in an ensemble, it would be important to report the range of input and output similarities in the ensemble. Are they always well constrained around the mean for all metrics and conditions?

5) Methodology to measure pattern separation:

5a) the type of activity pattern considered should be clarified as early as possible in the main text and/or figure 1. If I understand correctly, the method section makes this explicit, but it would help the reader to say in the result section that only temporal patterns are considered (i.e pairs of non-simultaneous spiketrains are compared, and the input similarity is the average over all spiketrains of the input ensemble). This is important because it helps comparing to other studies that have or have not considered the same type of patterns (e.g the Madar studies, as opposed to, say, Yim and Wolfart 2015 which considered spatiotemporal patterns). In particular, for the Hamming distance, this metric is usually used on population patterns rather than spiketrains. So, the results in Figure 1 are not directly comparable to the Myers studies or the Poirazi studies.

5b) Pattern separation levels are not measured as differences (Sinput – Soutput) but as ratios (Sinput/Soutput). This different from Madar et al. 2019 (to which the results are compared to) and to most past studies, and should be discussed. Could the authors explicit their rationale for that choice? I feel like Sin/Sout is not as easy to interpret as the difference. Especially if the similarity metric is not bounded between 0 and 1 like the Wasserstein metric. Details on the range of values for each reported metric and on how to interpret them would be useful.

5c) More details should be provided on the rationale behind the design choices for each sparsity-weighted information-theory metrics, as well as more details on how to interpret the values. Overall, I do not understand how sparsity, mutual information, transfer entropy and any combination of these can be considered measures of pattern separation, as these do not measure the similarity between spike-trains.

- Line 198: What is “differentiation” here? A synonym for separation? How is sparsity a similarity or separation metric?
- equation 1 shows that the "sparsity weight" is the difference of number of spikes normalized to the input number of spikes. That means it'd be negative if there are more spikes in the output: is that desirable?
- I also wonder why normalizing to the input sparsity (as opposed to an average of input and outputs, or their sum, or something else, if the goal is to make the metric less dependent on pairwise firing rates).
- I don't see how this sparsity-weight is a similarity metric or how it assesses pattern separation. As I understand it, sparsity is a feature of a spike-train, not a similarity measure. But one could design a similarity metric that would evaluate the dispersion (or entropy) of sparsity across multiple spike-trains, as in Madar 2019b. That is not what is done here, as far as I can tell.
- Line 200 “the simple sparsity measure is preferred as it makes no assumptions on the pattern completion circuit: not sure what is meant here. What assumptions do other measures make that sparsity doesn't?
- Line 200 “requires no discretisation of spike times”: if sparsity is simply the number of spikes, then there is a discretization choice in the length of the spike-train considered. The choice of sike-train length is as arbitrary as a bin size. Madar et al. 2019b consider such a measure as the “occupancy” feature of a spiketrain for a time bin as long as the total spike-train duration.

5d) Line 204-218: I am not convinced by the idea of multiplying similarity (or sparsity) measures with information transmission measures. Authors argue that they want to measure the “balance” between the two, but combining them makes the interpretation more difficult, as there are possible interactions. For instance, a situation with low redundancy reduction but high mutual info would give a similar value than a high redundancy reduction and low mutual info scenario, wouldn’t it? Providing more details on such cases would help the reader interpret the results throughout the manuscript. I personally think it's easier to interpret when separation and information transmission are not combined. The redundancy difference can be used as a very useful pattern separation metric. The mutual information can be evaluated separately.

5e) The neural codes assumed for information-theory metrics are not precisely defined. What was the word length, letter length, and other relevant parameters? These parameters should be somewhat justified. Is this where the authors chose the strategy of maximizing information, as suggested line 224? (I don’t see why this particularly more principled than using biologically relevant timescales or checking multiple timescales, as detailed below in point 6. We don’t know that the code is optimal).

5f) It would be interesting, and useful for interpretations, to explicitly show how the new metrics relate to classic pattern separation metrics (for now, we have Fig 3B and Fig S4 to compare). For example, Fig 3B (line 249-52) suggest that there's a correlation between the redundancy reduction measure and more traditional correlation measures (e.g. phase-locking strength, which would be picked up by Pearson’s R too), which is expected if it's really about redundancy. And there’s no correlation with the mutual info metric, which makes sense if it’s not based on similarity measures.

6) Line 156-157: This is debatable. The discretization choice is not necessarily arbitrary, some timescales are relevant for biological reasons: 10-20ms corresponds to membrane time constants, over which inputs can be integrated. 100-250ms corresponds to the theta rhythm which modulates entorhinal and hippocampal spike-trains. Multiple timescales are likely relevant and carry different meaning, so I think it is wise to check several of them. Given that the relationship between bin size and pattern separation levels is continuous, it's ok to check only a few. The bin size parameter is not a trivial issue but, in my view, it is not an intrinsic disadvantage of binned metrics compared to metrics based on raw spike times like the Wasserstein distance: it depends on how spike-trains are read by downstream targets. Raw spike times may be irrelevant in some cases. More likely, multiple timescales are relevant.

Moreover, as said in point 1b) above, information-theory metrics presented in this manuscript suffer similar issues of discretization choice. This is acknowledged in the methods (line 458) where it is mentioned that “the coding strategy and discretization should in principle be chosen to maximize information”. I don’t see why this is particularly principled, and why the same could not be done for classical metrics (i.e. finding the timescale that maximizes similarity).

8) Presentation of the results: In Fig 1 and 2, where multiple conditions, neural codes and similarity metrics are represented, it is hard to map the color shade to the legend. The graphs are dense in information, so increasing contrast between colors/symbols or separating plots would help.

9) Interpretation of the results: Throughout the manuscript, the interpretation of the results could be fleshed out a lot more, making more explicit what the results reveal about the meaning of each metric or about the modeled system (singe GC or DG network). Some examples:

- on line 163, what do the non-monotonic behaviors for the decorrelation and Wasserstein measures mean? Why do they occur?
- Line 222 “In all cases there is a single peak at an intermediate value of the thinning filters.”: Why? What does that mean?
- What do the peak of the curves in Fig 3B mean?
- The meaningfulness of Fig 3 and 5 results is not clear. More explicit interpretation would help the rea

10) Line 230: Why is gamma_M concluded to be the most useful pattern separation metric?

Since I don’t understand how gamma_T and gamma_M can be considered pattern separation metrics, that conclusion is far from obvious. The argument for them as pattern separation metrics and for how the authors rank the usefulness of each metric needs to be explicit.

Similarly, in the discussion (line 343) it is claimed that “We have demonstrated

our new techniques on single cell models and shown that they allow more useful conclusions to be drawn”. I disagree with the argument presented on line 329-39 (for reasons detailed above) and don’t see how gamma_R for instance is necessarily more useful than other classical measures. They may be complementary, as they make different assumptions on the neural code and measure different things. The argument for preferring the use of gamma_R (or gamma_M) over any other metric needs to be bolstered or the claim modified.

11) Figure 3:

a) 3A + line 241: what does each group of 5 input patterns represent? 5 different patterns at the same synapse, with a certain correlation structure? I imagine there are 4 groups for the 4 strong yellow synapses. Please clarify how these inputs were made and what synapse they are stimulating when.

b) 3B: what was the neural code considered?

c) “Spike traces are two minutes long and consist of phase-locked inputs with a phase rate of 0:6Hz and a spiking rate of 5Hz.” why those parameter choices? Do EC inputs show such a slow-frequency modulation and firing rates? From Desmukh and Knierim (2010) and Pernia-Andrade and Jonas 2014, there's a clear theta-modulation of EC inputs, seemingly mostly due to MEC inputs. Not sure about slow oscillations. LEC inputs don't seem to show much phase modulations, based on Desmukh and Knierim 2010.

12) Figure 4: It is useful to show how long the trains must be. An other graph, showing how much data is needed (how many spiketrains, how many repetitions, etc) to be able to compute accurately the exact values would help experimentalist design their experiments so that they can use those methods.

13) Line 285, Figure 4: How many spiketrains are considered in the input and output groups? Does the number of spiketrains being compared impact the accuracy of the exact MI calculation or the KL estimator? What about the correlation structure of the spiketrain ensemble: does it affect MI exact and estimated values?

14) “Pattern separation in a network” Line 289: Here, despite considering cells integrated in a network, pat sep is still evaluated at the single-cell level, considering only temporal patterns, correct? Or are spatiotemporal patterns (i.e. population of simultaneous spiketrains) considered?

15) Figure 5C-E: as for Figure 3, please clarify the neural code assumed and why this code was chosen.

16) Fig 5B: So, each of the five groups of GCs receives 1 of the input rasters displayed? The same raster is activating multiple synapses on each GC? Please clarify where and when input patterns are delivered.

17) Fig 5C: As pointed out above in comment #4, it's confusing to display different measures (one being correlation, the other redundancy reduction) for inputs and outputs. Why not just show redundancy for both inputs and outputs, on one hand, and show how redundancy reduction relates to decorrelation (or phase-modulation strength, in your case) on the other?

18) Line 302-5, Figure 5D: Since scaling the synapses strength up is done, why not show the effect of scaling down as well (using Jungenitz 2018 to constrain the parameter range), since you hypothesize its importance for pattern separation levels in abGCs.

19) Fig 5E and S5B: Both panels look surprisingly similar: Why? If I try to interpret the results (which the authors should expand on), it suggests that a pathological increase in intrinsic excitability, or in input strength, or both, leads to information loss (as assessed by the sparsity-weighted mutual info) and a decrease in pattern separation (as assessed by the redundancy reduction). This would be unexpected, since information-destructive unreliability generally improves better pattern separation (as shown in Fig 1). But, as pointed before (comment #5d), the similarity between 5E and S5B may simply result from the fact that the redundancy reduction metric is combined with the mutual information. So maybe the information loss is just driving the result in both figures here, not the redundancy reduction. This is why keeping separate the measure of pattern separation and the measure of information transmission is so important for interpretability. If the conclusion that indeed both pattern separation and information transmission are reduced in epilepsy, then more analysis to understand how epileptic alterations impair both aspects of dentate computations would be very interesting.

20) Is the network fully deterministic, or is neurotransmission stochastic like in the single cell model?

21) Line 582: The network lacks many important features, such as feedforward inhibition, interneuron diversity, and mossy-cell effects on inhibition and through their extra-laminar connections. Perhaps most relevant to the conclusion of the papers, the abGC connectivity is not very realistic (should be only weakly coupled to feedback inhib, cf Temprana and Schinder 2015). The network is also quite small. Because of these limitations, it’s not clear what meaning or weight to put in the results shown in Fig 5. These limitations should be acknowledged and discussed.

22) I understand that the goal is primarily to show how to use the new metrics in different settings and that the network simulated is for a proof of concept. But it would help the authors’ case to show how interesting conclusion can be drawn from their analysis. To that end, maybe it’d be useful to show how they could also be adapted to spatiotemporal patterns (e.g. like in Yim and Wolfart 2015 but with longer spiketrains)? (see comment #14 and 15). Actually, a direct comparison with Yim and Wolfart 2015 or Madar et al. 2021 would be interesting. They could more directly show in what way their new metrics adds value in comparison to past analyses.

23) Fig S2E-H: The densities don't match the scatter plots perfectly... What is the first value of similarity shown ? (e.g. in Fig S2G top left, there's a high density above 0.3 not seen in FigS2E. Similarly, density goes above 800 for bottom panel, but not in corresponding scatter plot)

**Minor comments:**

1) Author summary: “Traditionally, pattern separation has been described alongside the downstream networks that receive the separated representations” : Phrasing is not clear. I gather from the rest of the paper that it’s related to the argument about pattern separation being considered without reference to pattern completion.

2) Line 47: “increased”? Not clear what the measure is

3) Line 50: could cite Kreutz papers that introduced raw spike time metrics used in Madar et al. 2019b. Cutts and Eglen 2014 could be cited too, with their STTC metric on raw spike times.

4) Line 56 “later studies to use…”: grammar?

5) Line 123: could add other refs: O'reilly and McClelland 1994, SeveraAimone2016, Cayco-Gajic and Silver 2019, Madar et al 2019a and 2021).

6) Line 213-215 is an exact repetition of line 191-193

7) Line 214 “Here this is idea”: typo

8) Line 234-5: The phrasing is a bit misleading. The fact that single cells contribute to pattern separation is not in opposition to the role of circuit structure. The single-cell recordings in Madar 2019 were not made in isolation from the network and, in fact, the Madar studies show that pattern separation evaluated at the single cell level is not fully dependent on intrinsic cell properties but strongly dependent on network mechanisms (inhibition, short-term synaptic plasticity).

9) Line 291: I find the Hainmueller 2020 review confused on the matter of pattern separation. Mechanisms of pattern separation for population codes are best reviewed in Cayco-Gajic and Silver 2019. The Madar studies show experimental evidence for network mechanism at the single cell level.

10) By the way, in the bibliography, 2 different Madar et al 2019 are listed. These should be distinguished as 2019a and b in the main text, for clarity.

11) Figure 5D: The color code is confusing because it is the same as in panel C, where blue and grey correspond to differences in input frequency not input phase-modulation strength.

12) Lines 495 and 499: I am not an expert in information theory, but shouldn’t the number of words depend on the word length w? (r+1)^w ?
